# Distinct neuronal alterations distinguish two subtypes of sporadic Creutzfeldt-Jakob disease with shared dysfunctional pathways

**DOI:** 10.1172/JCI194721

**Published:** 2026-02-12

**Authors:** Katie Williams, Bradley R. Groveman, Simote T. Foliaki, Brent Race, Arielle Hay, Ryan O. Walters, Tina Thomas, Gianluigi Zanusso, James A. Carroll, Cathryn L. Haigh

**Affiliations:** 1 Division of Intramural Research, Laboratory of Neurological Infections and Immunity, and; 2 Rocky Mountain Veterinary Branch, National Institute of Allergy and Infectious Diseases, Rocky Mountain Laboratories, NIH, Hamilton, Montana, USA.; 3 Department of Neurosciences, Biomedicine and Movement Sciences, University of Verona, Verona, Italy.

**Keywords:** Cell biology, Infectious disease, Neuroscience, Prions

## Abstract

Prion diseases are a family of transmissible, neurodegenerative conditions caused by misfolded proteins called prions. Human cerebral organoids can be infected with prions from sporadic Creutzfeldt-Jakob Disease (sCJD) brain tissue. Initial experiments indicated that the cerebral organoids may be able to differentiate biological properties of different sCJD subtypes. If so, it would be possible to investigate the pathogenic similarities and differences. Herein, we investigated multiple infections of cerebral organoids with 2 sCJD subtypes, comparing hallmark features of disease as well as neuronal function and health. Our results show that, while all infections produced seeding-capable prion protein (PrP), which increased from 90–180 days after infection, a sCJD subtype preference for protease-resistant PrP deposition was observed. Both subtypes caused substantial electrophysiological dysfunction in the infected organoids, which appeared uncoupled from PrP deposition. Neuronal dysfunction was associated with changes in neurotransmitter receptors that differed between the subtypes but produced the same outcome of a shift from inhibitory toward excitatory neurotransmission. Further changes indicated shared deficits in mitochondrial dynamics, and subtype influenced alterations in intracellular signaling pathways, cytoskeletal structure, and the extracellular matrix. We conclude that cerebral organoids demonstrate both common mitochondrial deficits and sCJD subtype–specific changes in neurotransmission and organoid architecture.

## Abstract 2

Using a model of human brain tissue we have found that within the same family of dementia the brain may become damaged by different mechanisms.

## Graphical abstract

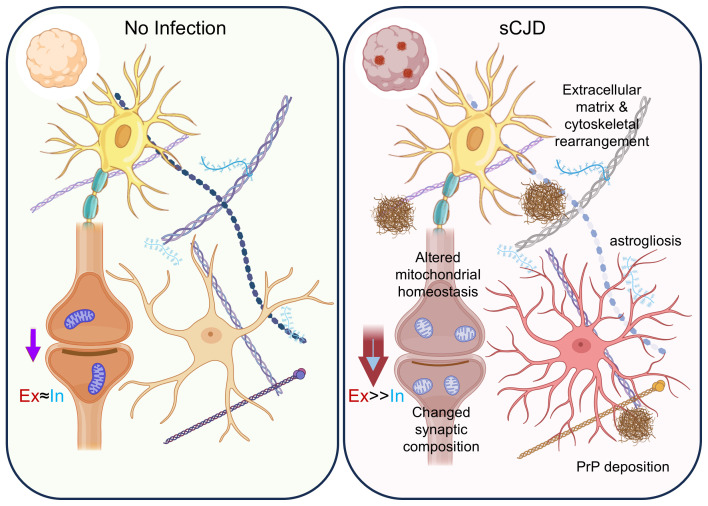

## Introduction

The prion disease family of neurodegenerative conditions can occur sporadically, arise due to hereditary mutation within the prion protein gene (*PRNP*), or be caused by infection from a contaminated source. Once formed or acquired, pathogenic isoforms (prions) can act as a template for seeding the formation of more pathogenic isoforms from the normal cellular prion protein (PrP^C^), and this templated formation of new prions propagates the disease throughout the brain. There are currently no effective treatments for this family of diseases, which are always fatal following symptom onset.

Clinical disease correlates with the development of hallmark features in the brain, including spongiform change, astrogliosis, and neuronal degeneration. As the disease progresses, prions accumulate in a typically proteinase digestion resistant isoform (PrP^Res^) and deposit in the brain. The most common human prion disease is sporadic Creutzfeldt-Jakob Disease (sCJD). Sporadic CJD has several subtypes, which have different clinical presentations and disease courses based on the affected brain regions (1). sCJD subtypes are classified according to 2 primary molecular features; the first distinguishes between types based upon the molecular migration of PrP^Res^ detected by Western blotting, and the second is determined by genotype at codon 129 of *PRNP*, which can be either homozygous for methionine (M/M) or valine (V/V), or heterozygous (M/V) (1, 2). Clinical presentation varies depending upon the subtype, which has traditionally been difficult to model in vitro (3).

Until recently, the primary difficulty hindering the study of human subtype pathology was a lack of human models capable of propagating sCJD prions. The first fully human model of infection to be developed used astrocytes generated from human induced pluripotent stem cells (iPSCs), which were obtained from donors with different codon 129 genotypes (4). The authors were able to demonstrate that the astrocytes would take up prion infection and showed PrP^Res^ with representative subtype mobilities, and that subtype preferences for the codon 129 genotype were preserved. In the second model to be developed, our group demonstrated that human cerebral organoids, three-dimensional spheres of human tissue containing neurons, astrocytes, and oligodendrocytes (5, 6), could also become infected and propagate prion infection (7). In this initial study, one inoculum each of 2 subtypes (MV1 and MV2) were studied and showed different accumulation and deposition in the organoids, indicative of a different response to the subtypes. The subtype-specific features of the infecting prions were shown to be preserved when the organoid prions were transmitted to humanized transgenic mice (8). However, as only one inoculum of each subtype was considered, conclusions on subtype-specific pathways of pathogenesis could not be drawn.

Herein, we aimed to examine the pathogenesis of the 2 sCJD subtypes (MV1 and MV2) in human cerebral organoids using multiple inocula per subtype and identify pathways with common shared changes as well as those that show subtype specificity. During human disease, MV1 prions display synaptic type deposition, whereas MV2 have plaque-like, focal PrP deposits (9). The subtypes also display differing clinical features, disease durations, and lesion profiles (9); thus, we hypothesized that different pathogenic pathways may be observed in the organoids. Our results show that organoid responses can differentiate the CJD subtypes based on the alteration of markers of infection and cellular pathways. Moreover, these data show a conserved functional impairment in neurotransmission, with changes in mitochondrial dynamics and the extracellular matrix, despite nuances between the subtypes.

## Results

### PrP^Res^ deposits more rapidly in MV2-infected organoids.

**Figure 1:**
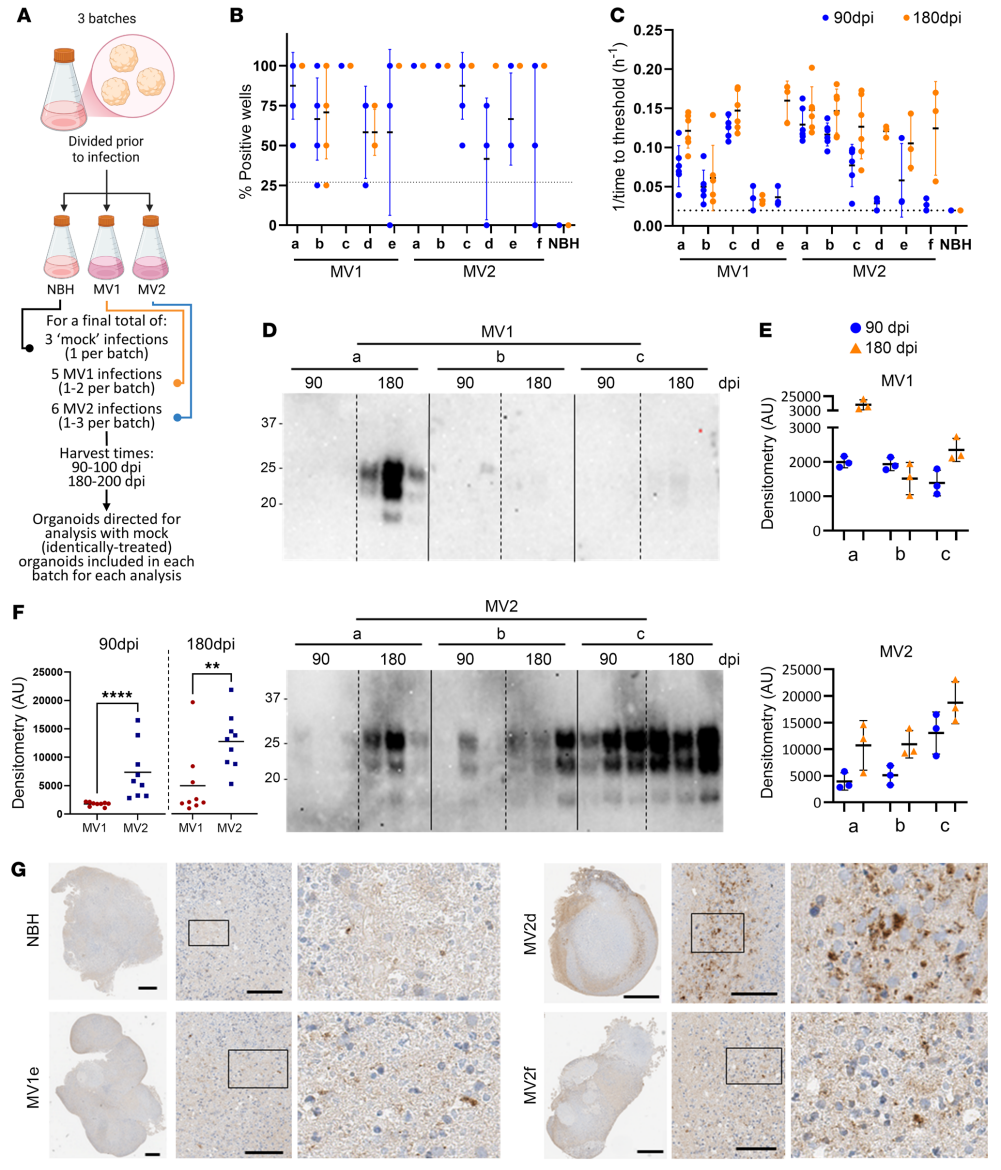
Prion infection of the cerebral organoids. (**A**) Schematic showing the experimental approach of multiple infections and organoid batches. Created with Biorender. (**B** and **C**) RT-QuIC analysis of the organoids at 90 (blue dots) and 175–180 (orange dots) dpi, showing the percentage of positive wells per reaction (**B**) and the reciprocal of the time to threshold (**C**). Dotted lines indicate the threshold above which the reaction is considered positive. (**D**) Western blotting following proteinase K digest for PrP of organoids harvested at 90 and 180 dpi. Each lane is from a single organoid. (**E**) Densitometric quantification of **C**. (**F**) MV1 and MV2 infection densitometry in **D** combined by infection. (**G**) Example PrP IHC images of the NBH and infected organoids. Scale bars: 500 μm for whole organoids;100 μm for 20× magnified fields. The boxed region is shown magnified to the right. On all plots, individual points indicate single organoids, **P < 0.01, ****P < 0.0001, Mann Whitney *U* test.

Human cerebral organoids were infected with 5 MV1 and 6 MV2 brain homogenates (Table 1) over 3 independent differentiations (batches), which each included a normal brain homogenate (NBH) control group that was cultured and harvested identically to the sCJD-infected organoids (Figure 1A). At 90- and 180-days postinfection (dpi), organoids were tested for parameters of prion infection. All infections showed RT-QuIC seeding activity, with most showing an increase in positive wells and/or a faster time to threshold at 180 dpi (Figure 1, B and C). Overall, the organoids infected with MV2 prions showed greater deposition of protease-resistant PrP that was detectable earlier at 90 dpi by Western blotting, with all MV2 infections showing some deposition at 175–180 dpi, as compared with the MV1-infected organoids where only 2 infections showed PrP^Res^ (Figure 1D and Supplemental Figure 1; supplemental material available online with this article; https://doi.org/10.1172/JCI194721DS1). In similarity with the results of the RT-QuIC, protease resistant PrP increased from 90 to 180 dpi for most infections (Figure 1, E and F). Some deposition of PrP could also be seen by histochemistry and was more apparent in the organoids infected with the MV2 subtype (Figure 1G and Supplemental Figure 1).

### Other hallmarks of prion infection.

Beyond disease-associated PrP accumulation and deposition, other hallmarks of prion disease include astrogliosis, spongiosis, cell death, and neuronal dysfunction. By 180 dpi, most organoids infected with the MV2 subtype and some with MV1 infections showed increased Glial Fibrillary Acidic Protein (GFAP) (Supplemental Figure 2). Spongiosis was not observed to be overtly increased over control organoids, as organoids have a background level of vacuolation that makes discerning disease-associated spongiform changes difficult. Example H&E staining at 175 dpi is shown in Supplemental Figure 3. No evidence of increased cell death was observed, and some protein and gene changes, including decreases in p53 and Bad in some infections, indicated that apoptotic pathways may be suppressed (Supplemental Figure 4). Thus, organoids become infected with and propagate human prions, demonstrating some of the hallmarks of disease but no toxicity in the timeframe investigated.

### Infected organoids display impaired neuronal function.

**Figure 2:**
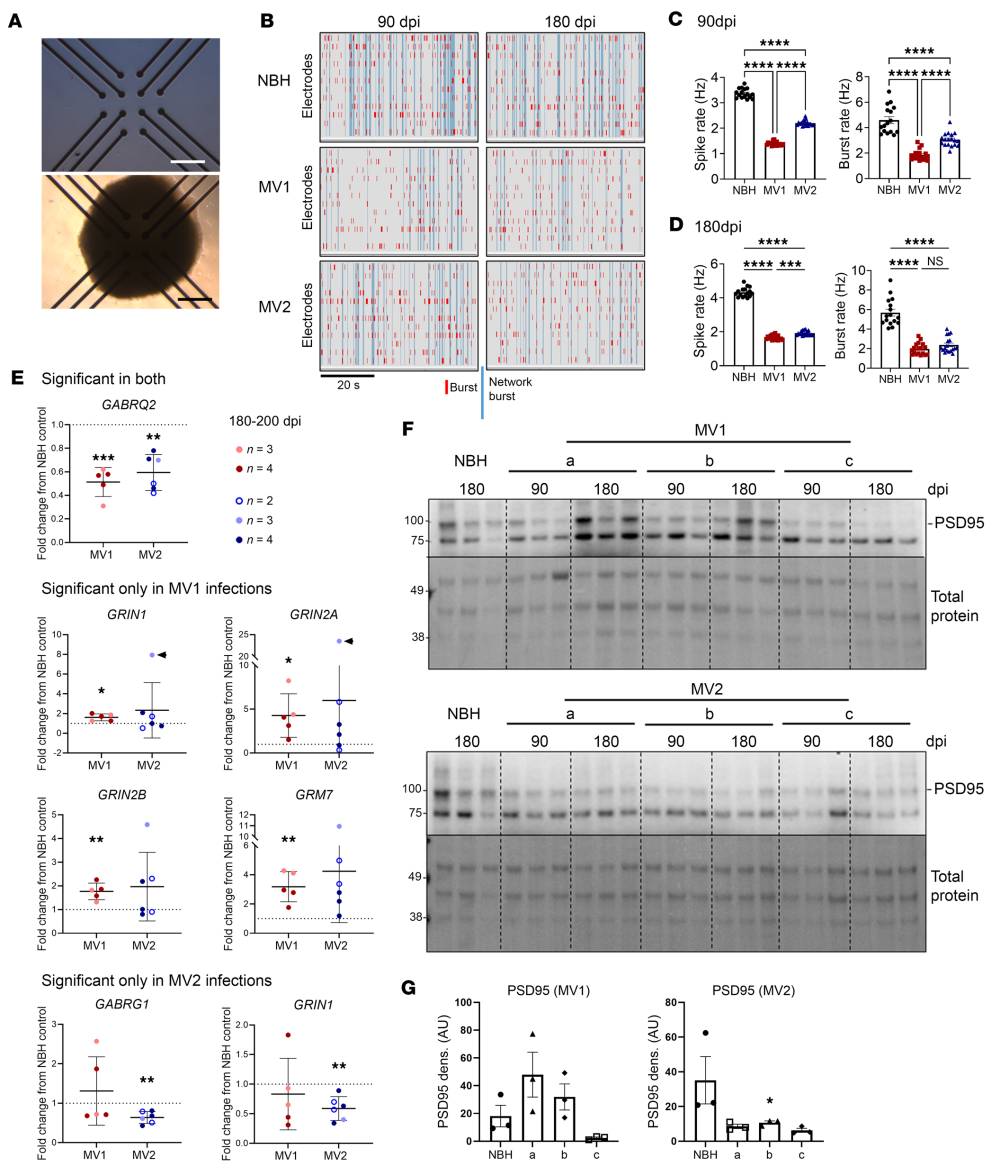
Neuronal dysfunction is a phenotype shared by MV1 and MV2 infected organoids. (**A**) Images of the multielectrode array (MEA) plates with and without an organoid situated on the electrodes. Scale bar: 1 mm. (**B**) Example MEA raw data collected. Organoid spike and burst rate at 90 (**C**) and 180 (**D**) dpi. Data shown combine 3 individual infections per subtype, each dot is a single organoid. (**E**) qRT-PCR analysis of neurotransmitter receptor genes shown as fold change from the NBH control (indicated by the dotted line; NBH *n* = 3 mock infections with 3–4 organoids sampled for each). Each dot is an individual infection, and the shading of the dot indicates the number of organoids sampled and averaged for that infection. Arrowheads indicate statistical outliers; removal of these from the analysis does not change the statistical test outcome. (**F**) Western blotting for PSD95, each lane is an individual organoid. (**G**) Densitometric quantification of the 180dpi PSD95 shown in **F**, each dot represents an organoid/lane. *P < 0.05, **P < 0.01, ***P < 0.001, ****P < 0.0001, Student’s *t* test (**E**), 1-way ANOVA with Tukey’s secondary test (**C**, **D**, and **G**).

To determine if the infections were influencing neuronal function, we used multielectrode arrays to measure neuronal electrophysiological function. Organoids were adhered to the electrodes as shown in Figure 2A for 48 hours and activity was measured as spikes (neuronal population action potentials) and bursts (local populations of neurons spiking synchronously). Network bursts where distal populations on different electrodes spiked together were also discernable (Figure 2B). By 90 dpi, the spike and burst rates of the infected organoids were significantly decreased, with the more pronounced deficit observed for the MV1-infected organoids (Figure 2C). By 180 dpi, equivalent decline is seen for both subtypes (Figure 2D). The data shown here include 3 independent infections of each subtype (A–C, individual responses are shown in Supplemental Figure 5), the remaining infections were subject to similar analysis but allowed to adhere for the duration of the infection to the electrodes. These infections showed the same results despite the different culture conditions (Supplemental Figure 5). To investigate the cause of the dysfunction, we performed qRT-PCR on a panel of neurotransmitter-receptor genes. Only one transcript change was shared by both subtype infections, a decrease in Gamma-Aminobutyric Acid (GABA) receptor subunit θ (Figure 2E, Table 2, and Supplemental Table 1). This subunit is part of the GABA A inhibitory signaling complex. Eleven further transcripts were significantly changed in the MV1 infections and included elevation of several genes associated with glutamatergic excitatory neurotransmission. Only 2 further transcripts were significantly changed in the MV2 infections, and both were decreased and associated with GABAergic inhibitory neurotransmission. Western blotting for PSD95 (a postsynaptic density scaffolding protein) showed variable detection in the MV1-infected organoids but generally reduced detection in the MV2 infections (Figure 2, F and G). Few changes showed any correlation with PrP seeding or deposition (Supplemental Figure 6), instead supporting substantially perturbed neurotransmission during infection, with differences in specific changes between subtypes, uncoupled from PrP deposition.

### Transcripts associated with mitochondrial fission-fusion dynamics are changed during infection.

**Figure 3:**
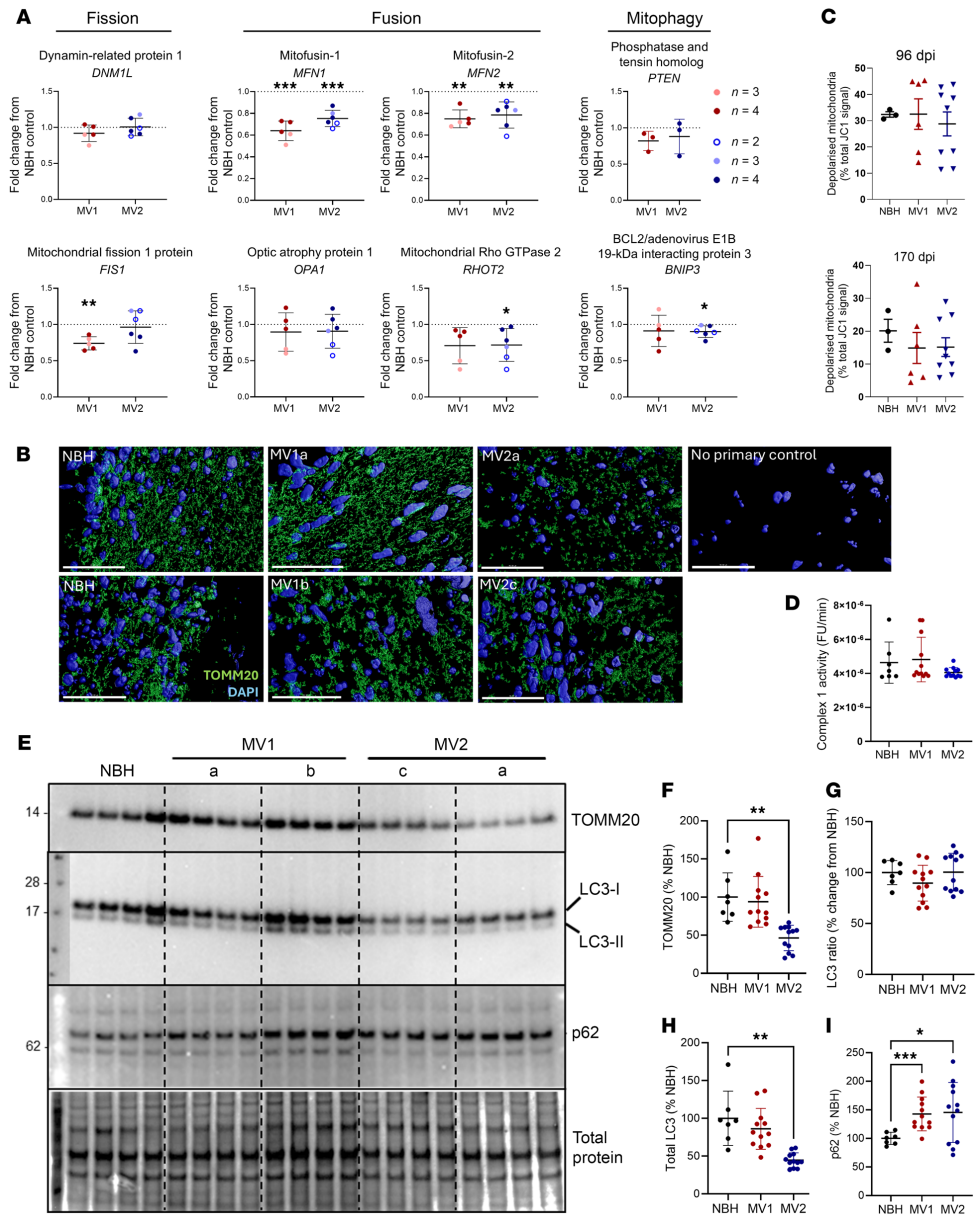
Organoids have reduced mitofusin transcripts but unaltered mitochondrial function and changed autophagy proteins. (**A**) qRT-PCR analysis of mitochondrial fission, fusion, and mitophagy genes at 180–200 dpi shown as fold change from the NBH control (indicated by the dotted line; NBH *n* = 3 mock infections with 3–4 organoids sampled for each). Each dot is an individual infection, and the shading of the dot indicates the number of organoids sampled and averaged for that infection. (**B**) Example morphology of mitochondria at 180 dpi labelled with TOM22 and DAPI nuclear stain in several sections imaged by confocal microscopy. Scale bar: 40 μm. (**C**) Mitochondrial inner membrane polarization determined by JC1 fluorescence at 96 and 170 dpi from 2 MV1 and 3 MV2 infections. Each dot is an individual organoid. (**D**) Complex 1 activity assay at 180 dpi from 3 MV1 and 3 MV2 infections. Each dot is an individual organoid. (**E**) Western blotting for TOMM20 at 180 dpi, each lane is an individual organoid. (**F**) Quantification of **E** plus one more infection per infection (see Supplemental Table 5 and uncropped Western blots), each dot is an individual organoid. (**E**) Western blotting for LC3 and p62 at 180 dpi, each lane is an individual organoid. (**F**–**I**) Quantification of TOMM20 (**F**), LC3 ratio (**G**), total LC3 (**H**), and p62 (**I**) plus one more infection per infection (see Supplemental Table 5 and uncropped Western blots), each dot is an individual organoid. *P < 0.05, **P < 0.01, ***P < 0.001 Student’s *t* test (**A**) or 1-way ANOVA with Tukey’s secondary test (**C**, **D**, **F**, and **H**–**J**).

Neurotransmission requires mitochondrial function, as mitochondria are central for energy production, neurotransmitter synthesis, and calcium balance (10, 11). Changes have been found within mitochondria in both animal models of prion infection and postmortem human brain tissue (12–15). We examined a panel of mitochondrially associated gene transcripts by qRT-PCR. Here, changes in 13 transcripts were shared between the infections with the 2 subtypes and all were decreased (Table 2 and Supplemental Table 6). Of note, several genes associated with fission-fusion dynamics were changed, including both mitofusins (Figure 3A), which may result in increased mitochondrial fragmentation. However, transcripts associated with mitophagy, including PTEN — which activates PTEN Induced Kinase 1 (PINK1), a known mediator of mitophagy — and BNIP3 were unchanged (Figure 3A). No overt changes in mitochondrial morphology were observed (Figure 3B), although, due to the heterogeneous nature of unguided brain organoids, imaging can miss regions of interest if they were not captured in the tested section. Measures of mitochondrial function, including polarization and complex 1 activity, were not different from NBH controls (Figure 3, C and D); however, polarization did become highly variable in the infected organoids. Western blotting for TOMM20 did reveal a decreased detection in the MV2 infected organoids that might indicate a reduction in total mitochondria, and this could occur if increased fission was permitting faster mitophagy. LC3 detection, a protein involved in autophagy, was also decreased in the MV2 infections, albeit without a change in the ratio of LC3-II to LC3-I (Figure 3, E–I). A decrease in LC3 might indicate increased autophagosome turnover and, therefore, turnover of LC3 or, conversely, reduced autophagosome formation (16, 17). Detection of the selective autophagy adaptor protein p62 was increased in both MV1 and MV2 organoid infections (Figure 3, E and I). Increases in p62 are generally considered to be indicative of inhibition of autophagy. Together, these data indicate that mitochondrial fission-fusion dynamics may be changing, but, while this could result in mitophagy, autophagy may be defective.

### Signal transduction pathways show perturbation.

**Figure 4:**
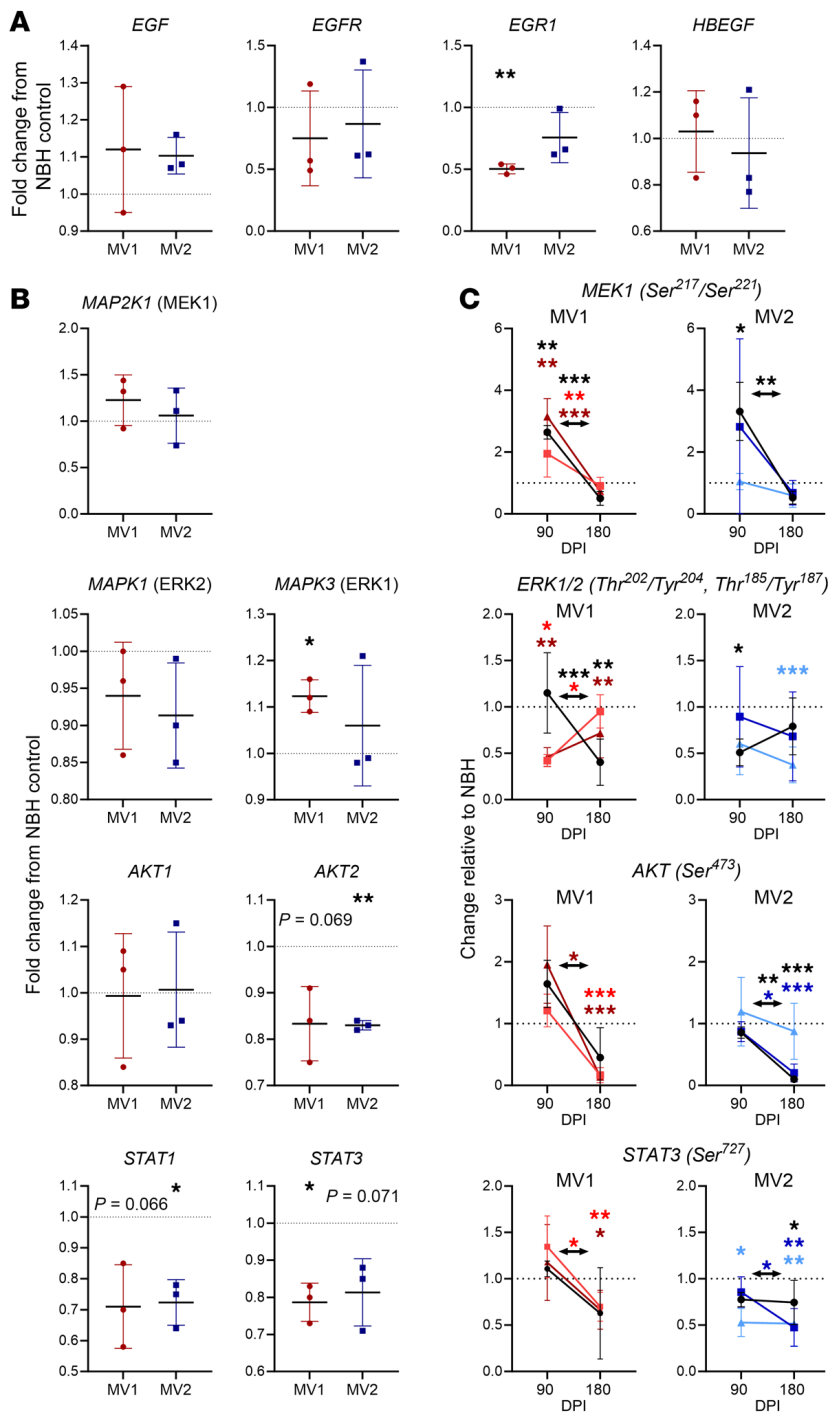
Changes in MAPK, AKT, and STAT signaling pathways are present during infection. (**A**) qRT-PCR analysis of gene transcripts associated with the EGFR signaling pathway at 180 dpi. Each dot is an individual infection for which 4 organoids were sampled and averaged, shown relative to the NBH control. (**B**) qRT-PCR analysis of gene transcripts associated with MAPK, AKT, and STAT signaling at 180 dpi. Each dot is an individual infection for which 4 organoids were sampled and averaged, shown relative to the NBH control. (**C**) Bioplex detection of phosphorylated signaling proteins at 90 (*n* = 3) and 180 (*n* = 6) dpi relative to the respective time point NBH control. Shown are the mean and standard deviation, lines/colors indicate the same infection sampled at each time point. *P < 0.05, **P < 0.01, student’s *t* test (**A** and **B**), 2-way ANOVA with Dunnett’s secondary test (**C**, colors indicate the significant infections). NBH controls are indicated by the dotted lines.

In our previous work looking at PrP function, mitochondrial changes were linked with EGFR signal transduction (18). Therefore, we assayed a panel of signaling protein genes by qRT-PCR to look for changes in this pathway. Upstream EGF-signaling associated transcripts were unchanged by infection at 180 dpi (Figure 4A), and Western blotting confirmed unaltered phospho- or total EGFR (Supplemental Figure 7), but a downstream transcription factor, Early Growth Response 1 (EGR1), was significantly reduced in the MV1 infections (Figure 4A and Supplemental Table 1). Examining other signaling intermediates showed that no changes in transcripts were shared by the MV1 and MV2 infections (Table 2), but changes were measured in MEK-ERK, AKT, and STAT signaling transcripts in one infection subtype, with most being decreased (Table 2 and Figure 4B). Examination of phospho-protein levels confirmed that most, but not all, signaling intermediates showed decreased detection at 180 dpi and indicated that protein levels were changed from 90 dpi, where some intermediates were increased (Figure 4C and Supplemental Figure 7). Thus, signal transduction pathways are influenced during infection without any clear subtype coherence and may be changed in a temporal manner.

### Matrix and cellular structure are changed in MV2 infections.

**Figure 5:**
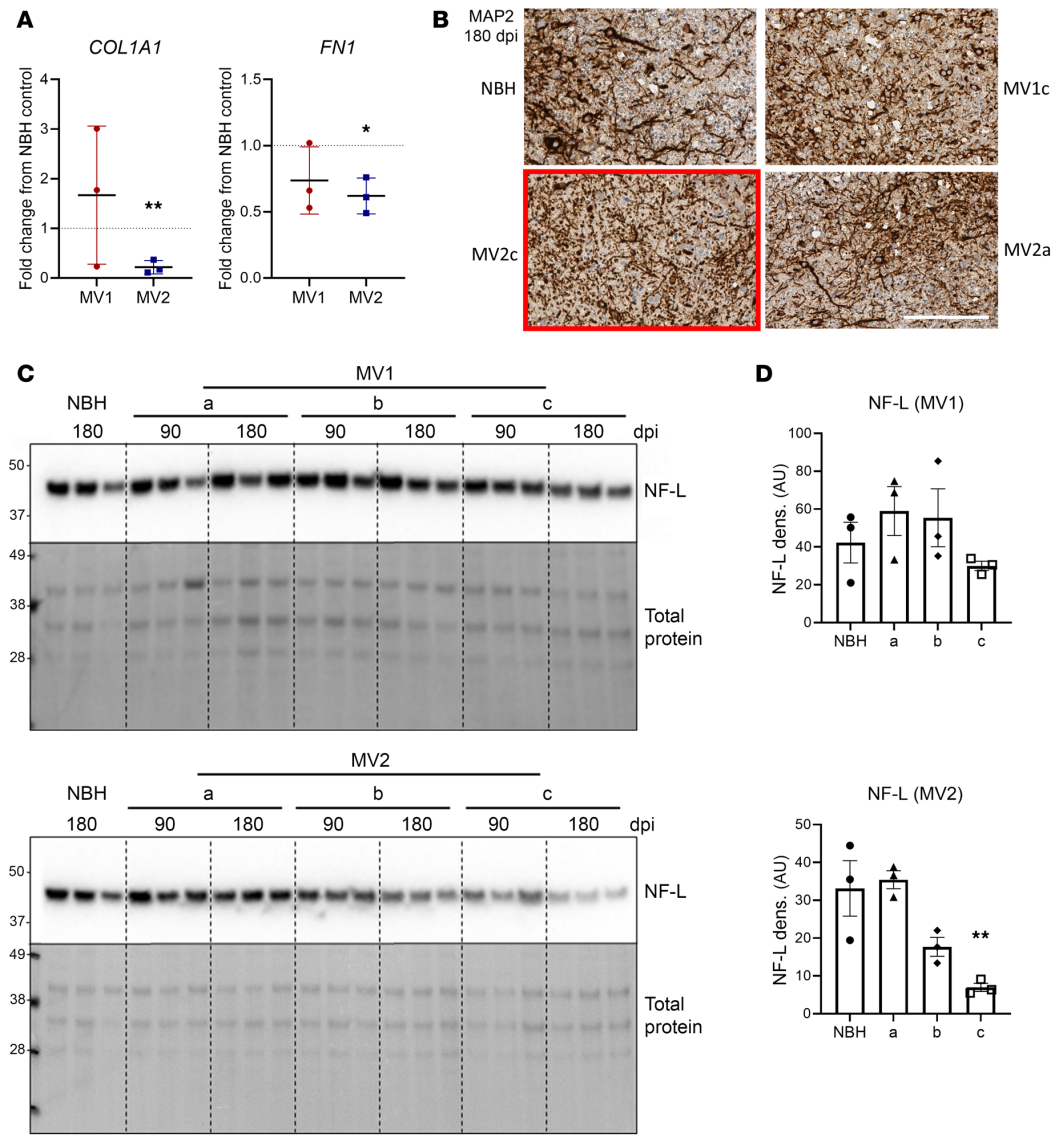
MV2-infected organoids show extracellular matrix breakdown. (**A**) qRT-PCR analysis of collagen and fibronectin gene transcripts at 180 dpi. Each dot is an individual infection for which 4 organoids were sampled and averaged shown relative to the NBH control (indicated by the dotted line). (**B**) Example MAP2 staining showing altered morphology in some regions of MV2 infected organoids (red box). Scale bar: 200 μm. (**C**) Western blotting for neurofilament-light (NF-L) chain. Each lane is a single organoid. Please note that the total protein stain is the same as that shown in Figure 2F, as multiple probes of the same membrane were performed. (**D**) Densitometric quantification of NF-L in C at 180 dpi, each point is a single lane/organoid. *P < 0.05, **P < 0.01, students *t* test (**A**), 1-way ANOVA with Dunnett’s secondary test (**D**).

A further observation from the EGFR gene panel was that collagen and fibronectin transcripts were significantly reduced in the MV2-infected organoids, indicating a potential disruption of the extracellular matrix (Figure 5A). Examination of microtubule-associated protein 2 (MAP2) labeling of sections from the organoids showed that, in some MV2 sections this staining was especially punctate, indicating altered neurite structure. Examples of this punctate staining as well as more normal sections that were observed for both infections are shown in Figure 5B. Another structural protein, neurofilament light chain (NF-L), was also shown to be significantly decreased in one MV2 infection (Figure 5, C and D). Together, this may indicate that the extracellular environment and its support for neurites has begun to break down in the MV2 infected organoids.

### Single cell RNA-seq identified changed cellular canonical pathways.

**Figure 6:**
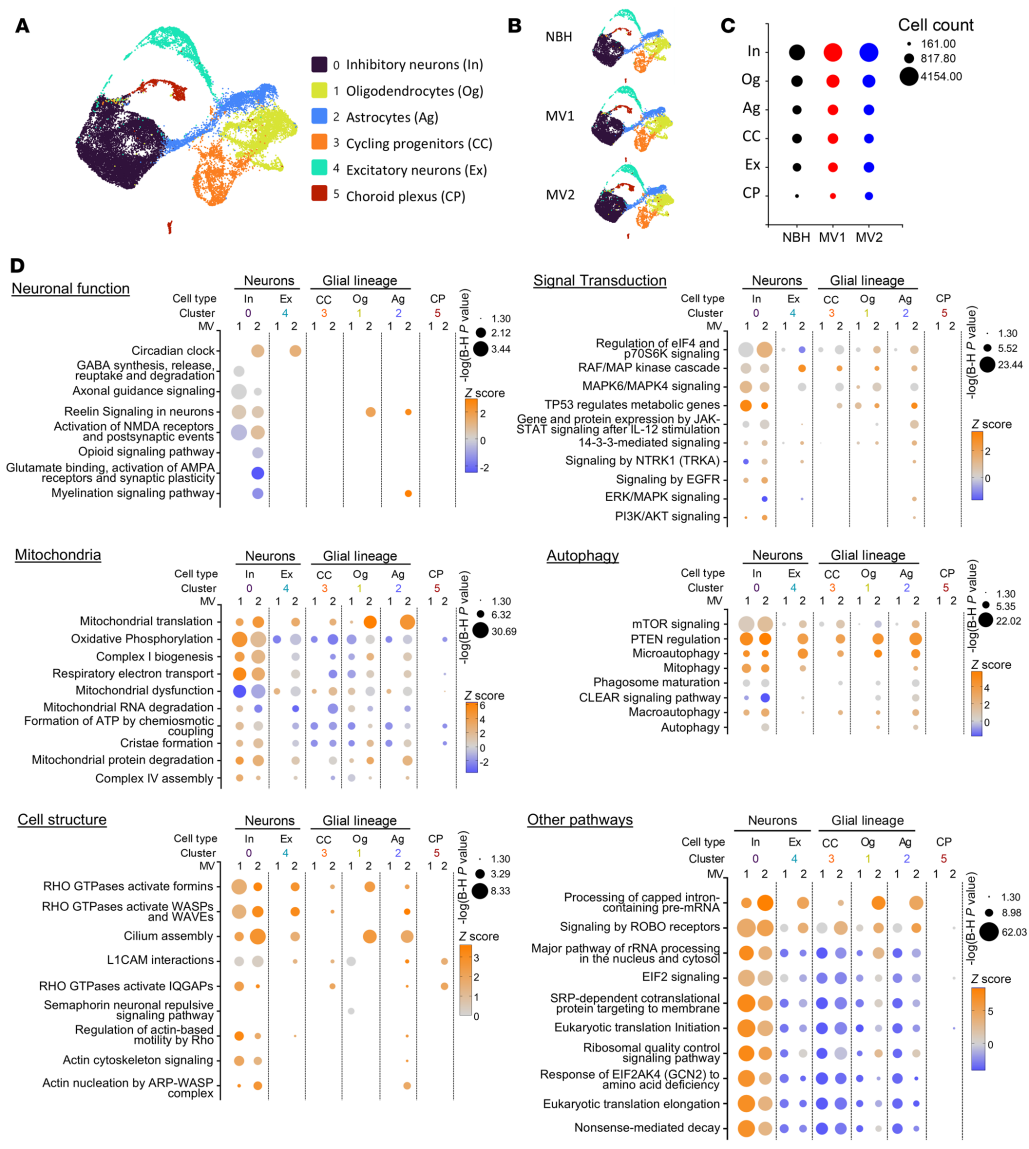
Single cell sequencing reveals changes in canonical pathways at 120 dpi. (**A**) Uniform Manifold Approximation and Projection (UMAP) of cell clusters, with their identification and split by group (**B**). (**C**) Number of cells per identified cell cluster for each group. (**D**) Ingenuity pathway analysis (IPA) showing changed canonical pathways across each cell type and infection when compared with NBH controls. The size of the node denotes the –log(B-H *P* value) where the lowest value is set to 1.30 (*P* = 0.05), thus, only conditions that met the threshold for significance have visible nodes, and the color indicates the IPA prediction of pathway activation (orange), deactivation (blue), or neutral/no calculated activity pattern (gray). Shown are up to 10 most significantly changed pathways relating to neuronal function, mitochondria, autophagy, cell structure and other highly significant pathways. Signal transduction pathways related to the data in Figure 4 and Supplemental Figure 7 were selected for presentation. Complete data can be found in Supplemental Table 3.

To further investigate how the infections were influencing organoid function, we performed single cell RNA sequencing (SCS) on 3 NBH, 4 MV1, and 3 MV2 infections from 2 separate batches with 3 organoids pooled per reaction. Organoids were assayed at 120 dpi, after all infections showed prion accumulation/seeding activity (example RT-QuIC reactivity for cells used in the SCS reactions are shown in Supplemental Figure 8, A and B) and neuroelectrophysiological dysfunction. We identified 6 clusters of cells (Figure 6A and Supplemental Figure 8C). The clusters followed mostly the same pattern of representation across the groups despite some variance in cell populations across infections (Figure 6, B and C, and Supplemental Figure 9) and PRNP detection was unchanged across infections (Supplemental Figure 10).

Pathway analysis of the differentially expressed genes (DEGs) was undertaken for all cell types per infection relative to NBH controls using Ingenuity Pathway Analysis (IPA) core analysis, then these were compared across cell types using comparison analysis. IPA identifies pathways with statistically significant gene changes and predicts from those changes whether the pathway would be activated (increased z-score) or deactivated (decreased z-score). No significant pathways were identified in the MV1 choroid plexus, as only 4 statistically changed genes (*MEA1*, *BEGAIN*, *PTGDS*, and *NDUFA1*) were identified. Using a significance cutoff of –log(B-H *P* value) = 2, 377 canonical pathways, and 68 diseases and biofunctions were identified as changed in one or more cell groups of each infection (full data in Supplemental Tables 2 and 3). The most changes in a cell type were seen for the inhibitory neurons, although this is most likely a reflection of these being the most represented cells.

We used IPA to examine pathways associated with neuronal function, signal transduction, mitochondria, autophagy, and cell structure for changes (Figure 6D). In agreement with our previous results, substantial changes in pathways associated with mitochondrial function were observed. Interestingly, for both infection subtypes, the inhibitory neurons showed changes indicating increased mitochondrial activity, but, in other cells, suppression of activity was predicted. The MV2 cell groups indicated increased activation of autophagy pathways possibly leading to the increased changes observed in autophagy proteins for this subtype (Figure 3, G–I). As we observed in our qRT-PCR data and Bioplex assays at 90 dpi, relatively few changes were predicted in signal transduction pathways at the 120 dpi SCS harvest time, and most of those predicted activation. Few changes were observed in pathways associated with neuronal function, and these were mainly within the inhibitory neurons. A few pathways were identified as significantly changed in only one subtype or changing in different directions between the MV1 and MV2 infections. As seen for the IHC analyses, some changes in cytoskeletal structure signaling pathways were also predicted for both infections in both the canonical analysis (Figure 6D) and the diseases and biofunctions analyses (Supplemental Table 3 and Supplemental Figure 11). Finally, of the other pathways that were changed, a number included processes involved in RNA transcription, translation, and processing (Figure 6D and Supplemental Figure 11). For these categories, there were some interesting differences between the inhibitory neurons and the other cell types, with pathways that were predicted to be activated in the inhibitory neurons also predicted to have reduced activity in other cell types (Figure 6D). Overall, the SCS analysis supported that changes were occurring within the pathways examined in our analyses herein but also indicated new directions that might be worthy of future study.

## Discussion

**Figure 7:**
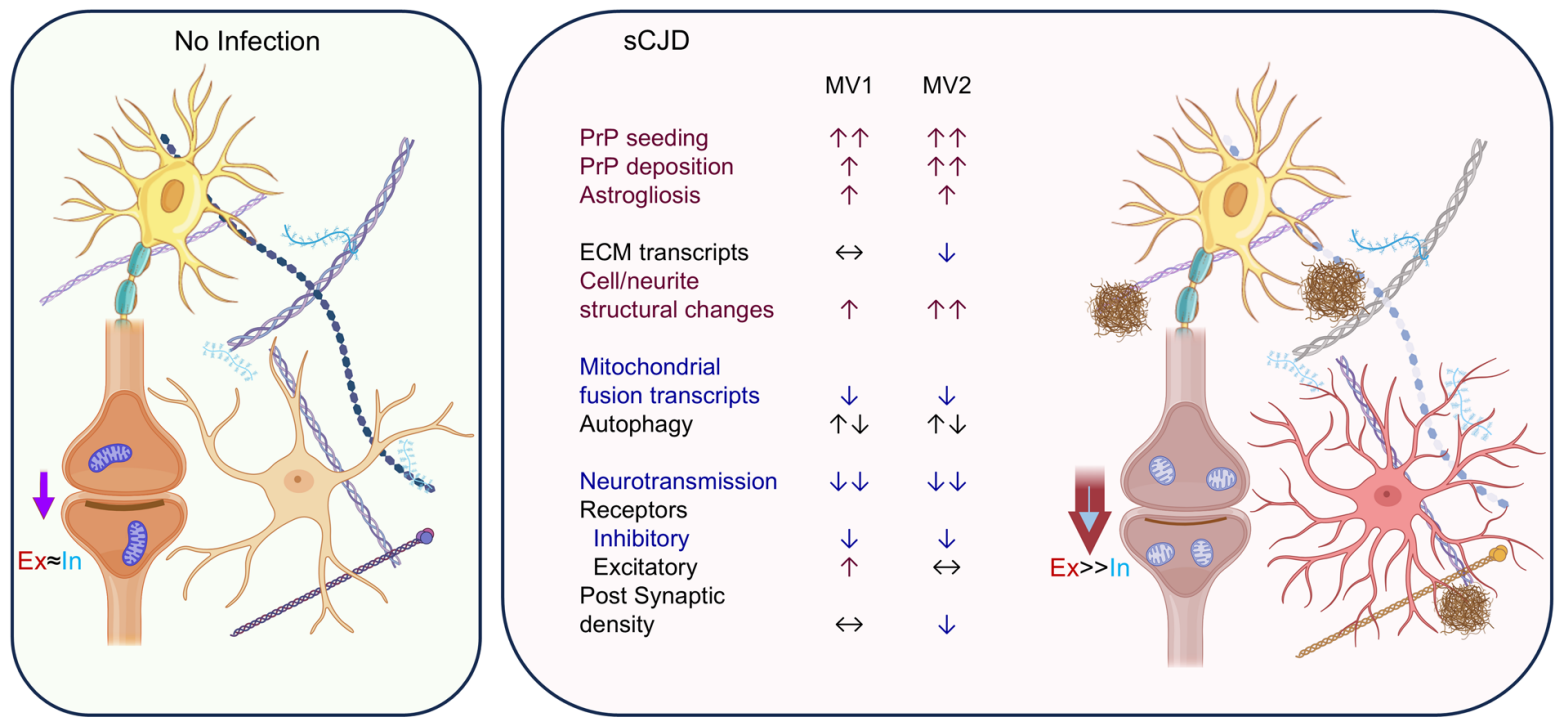
Schematic diagram depicting the changes occurring during organoids infections. General summary of cellular processes identified as changed during infection of the organoids with MV1 and MV2 sCJD prions. ↑↑, most/all tested parameters increased; ↑, some tested parameters increased; ↓↓, most/all tested parameters decreased; ↓, some tested parameters decreased; ↔, no change; ↑↓, some parameters indicating increased activation and some indicating decreased; Ex, excitatory; In, inhibitory. Created with Biorender.

Our previous studies showed that human cerebral organoids become infected with sCJD prions retaining the features of the original infecting inoculum (7, 8) and, therefore, they have the potential to be a useful model for investigating subtype-specific changes. Herein, we used multiple infections of cerebral organoids with 2 different sCJD subtypes, MV1 and MV2, to examine the similarities and differences between the infection phenotypes. In agreement with our previous work (7), the MV2 infections showed greater deposition of PrP. All infections of both subtypes showed early (90 dpi) deterioration of neuroelectrophysiological function, which was more pronounced in the MV1 subtype infections. Gene transcript analysis both by qRT-PCR and SCS indicated changes in neurotransmitter receptor subunits/membrane channels, and structural components of the axons, dendrites and extracellular matrix may all be contributing to these changes. Further changes were seen in the transcripts of genes associated with mitochondrial dynamics. While the changes measured affected similar pathways, differences in how these changes mediated their effect were apparent between the 2 subtypes. A summary schematic of the infection changes can be found in Figure 7.

Neuronal dysfunction has been reported in animal models of prion disease (19–23) and early functional impairment was associated with cytoskeletal breakdown and abnormal synapses (24). Across all infections, organoids showed electrophysiological decline. The composition of the neurotransmitter receptors supported a shift away from inhibitory GABAergic neurotransmission toward excitatory glutamatergic that appears more pronounced in the MV1 infected organoids, and is supported by shifts previously identified in human models of PrP E200K mutation (25, 26). Sporadic CJD subtypes MV1 and MV2 show major clinical and pathological differences, especially in hyperexcitability of cerebral cortices. For instance, in the MV1 subtype, myoclonus and atypical EEG (with periodic sharp and slow-wave complexes) are common findings, while in MV2, myoclonus appears late in the disease course, and the typical EEG tracings are rarely observed (9). The delayed pace of dysfunction in the MV2-infected organoids appears to parallel these changes. From our current data we cannot determine whether GABAergic neuronal dysfunction is responsible for shifting neuronal behavior toward an excitotoxic phenotype or whether excitatory neuronal changes themselves contribute. Evidence exists that supports both possibilities; one of the earliest populations to be selectively lost in CJD is the GABAergic interneurons, corresponding with the initial tissue lesions (27). However, a recent report also showed that prion propagation is more efficient in excitatory than inhibitory neurons (28). Altogether, considering both human patient and organoid findings, a putative shift away from inhibitory neurotransmission toward excitatory could result in neurotransmission defects that are more pronounced and/or occur earlier in the MV1 subtype.

The loss of GABAergic neurons in CJD is associated with early loss of the extracellular matrix (ECM) perineuronal nets surrounding these cells (29). A consequence of this may be further synaptic dysfunction, as the ECM has been shown to regulate GABAergic neurotransmission in the hippocampus (30). Herein, more pronounced changes in the ECM were evident in the MV2 infections and may be related to the more overt MV2 prion accumulation observed. Interestingly, fibronectin 1 (FN1) has been implicated in the susceptibility of cells to prion infection (31, 32), and silencing *FN1* expression significantly increased the rate of prion propagation (31). The reduced transcript levels observed in the MV2 infections may therefore be contributing to the increased accumulation of deposition of PK-resistant PrP. The ECM serves to critically support synaptic function (33, 34). Thus, not only may synapse composition be responsible for dysfunctional neurotransmission, but the structural changes to the ECM may hinder neuronal network communication while creating a more permissive environment for prion propagation.

The ECM is additionally crucial for transduction of signals from the outside environment into the cell (35). Herein, corresponding with the changes in the ECM, we found alterations in pathways associated with extracellular matrix signaling including MEK/ERK, AKT, and STAT pathways, all of which are signaled through the ECM-associated Src kinase family (35). These pathways have previously been found changed during prion infection in cell cultures and mice, mostly showing increased activation (36–40). Less is known about the signaling changes that occur in human disease, but studies examining postmortem CJD tissue have found preserved phosphorylated ERK levels in the frontal cortex by Western blotting (41), and, by histochemical analyses, diffuse increases in MEK/ERK detection in the cerebellum (42) and an absence of phosphorylated JNK (43). Dysregulation of these central cellular signaling pathways could have many consequences within the brain as they influence most cellular processes including transcription and translation, which were variably changed in the different cell types.

Examination of postmortem brain tissue from patients with sCJD has found numerous mitochondrial changes, including deficiency of the electron transport chain enzymes (12, 14), decreased mitochondrial DNA copy number (44), and altered mitochondrial gene transcripts (12, 45–48). For both sCJD subtype infections examined, decreases were seen in the mitochondrial fusion protein transcripts MFN1 and MFN2. This shift in balance away from fusion would likely increase fission. Fission can be associated with mitophagy, which was previously linked with disease progression (49). Western blotting for autophagy markers, particularly p62 indicated that it may be stalled. This might account for the increase in predicted activation of related pathways, including mitophagy, as cells attempt to compensate. In turn, activation of mitophagy, even if inefficient, could result in the predicted changes in pathways associated with mitochondrial protein synthesis and turnover. MFN2 has been demonstrated to be involved in axonal transport of mitochondria (50, 51). Certain MFN2 mutations are associated with loss of function in Charcot-Marie-Tooth disease (CMT), resulting in impaired mitochondrial trafficking along axons, which promotes degeneration (52). Potentially, if the un-compensated down-regulation of the MFN genes seen in our current study is prolonged, detrimental damage may occur within axons and dendrites as well as impairing the efficiency of ATP production through mitochondrial depletion.

Although limitations of the organoid model have been discussed previously (53), there are a number of important caveats to consider, specifically in the interpretation of the data herein. One specific caveat is that the organoids do not contain microglia or endothelial cells; and the absence of these cells may influence neuronal and astrocytic responses, which has been demonstrated for microglial ablation in mice (54). We might expect that if microglia were present, a more substantial neuroprotective response may be observed. Microglial ablation studies in mice agree that disease is accelerated in the absence of these cells and that reactive astrogliosis is enhanced, although they disagree on the role of microglia in prion clearance (55–57). In the context of the organoid model, disease may be accelerated due to the lack of microglial neuroprotective functions. Conversely, prions have been shown to play a role in the breakdown of the endothelial cells forming the blood brain barrier in association with neurotoxic astrocytes (58). Additionally, caspase activation was measurable in blood vessels of prion-infected mice (59). Taken together, this might cause dysregulated nutrient exchange. Since the organoid model does not have vascularization and nutrients are regularly replenished/waste removed, culture conditions may be favoring the survival of the organoid cells in a way that does not happen in vivo. Despite these limitations, many of our observations in the organoids are in agreement with those seen across other models; for example, as discussed above, perturbation of neuronal function, mitochondria, and the cytoskeleton have previously been reported in prion diseases. Our observations add valuable information about the human-specific changes affecting neuronal populations in different sCJD subtypes.

### Conclusions.

Infection of human cerebral organoids with 2 subtypes of sCJD showed changes in neurotransmission that could be caused by shifts in receptor composition, mitochondrial dynamics, and cell structure/ECM remodeling. Further, the changes differ between the sCJD subtypes, despite producing the same neuronal dysfunction outcome. Prion strain properties might have a role in explaining the differences in pathology. The more pronounced early neuronal dysfunction in MV1-infected organoids may be related to the synaptic tropism of PrP deposition, potentially causing neuronal damage. In MV2 infection, the more pronounced changes associated with the extracellular matrix would be related to the more prevalent PrP extracellular plaque-like deposits. The aforementioned neuronal molecular events in human disease are recapitulated in our human cerebral organoid model. Since sCJD brain tissue is observed in the final stage of the disease, investigations are limited by RNA and protein degradation. Thus, the human cerebral organoid model offers the ability to address early phases of the disease process that cannot be easily studied in human brain tissue and potentially provides insights for development of therapeutic intervention strategies.

## Methods

### Sex as a biological variable.

Sex was not considered as a biological variable in this study.

### Human-induced pluripotent stem cells and culture.

KYOU-DXR0109B (ACS-1023; ATCC) hu-iPSCs were routinely cultured on low growth factor Matrigel (Roche) in mTeSR1 medium (Stem Cell Technologies) with 5% CO_2_ in a humidified incubator as described in the mTeSR handbook. Colonies were passaged at approximately 70%–80% confluency before colonies had started to contact each other.

### Organoid generation and culture.

Cerebral organoids were generated using a cerebral organoid differentiation kit (Stem Cell Technologies) and cultured as described in ref. 7, based on the original protocol described by ref. 6. Three separate batches (differentiations) of organoids were used in this study to avoid batch variability as a confounding factor. Organoids from the same batch were separated into test groups at the time of inoculation. Every batch contained a normal brain homogenate (NBH; brain tissue from a donor who died from a non-CNS associated condition) control group that was treated and harvested at the same time as the sCJD infections. For long term culture, organoids were maintained in cerebral organoid media (1× GlutaMAX, 1× penicillin-streptomycin solution, 0.5× nonessential amino acids, 0.5% [v/v] N2, 1% [v/v] B12 plus retinoic acid, 1 μL/4mL insulin, and 1 μL/286 mL 2-Merceptoethanol in 1:1 Neurobasal:DMEM-F12 medium) at 37°C in a standard CO_2_ tissue culture incubator with agitation at 65 rpm. Media was changed weekly or as required.

### Prion infection.

Brain homogenates from sporadic CJD subtypes MV1 and MV2 (described in Table 1 and Supplemental Figure 12) were diluted into organoid maintenance media to a final concentration of 0.1% (tissue wet weight/volume). One homogenate each was include in 2 separate batches to check reproducibility. At the start of infection, existing media was removed from the organoids and replaced with the inoculated media. Twenty-four hours after inoculation an equivalent volume of fresh media was added to the cultures (diluting the original inoculum 1 in 2). A full media and culture vessel exchange was performed 7 days after initial exposure. Organoids were maintained in agitated culture with media changes weekly.

### RT-QuIC.

Real-time Quaking Induced Conversion (RT-QuIC) assays were performed similarly to those reported previously (7, 60). Briefly, the RT-QuIC reaction mix contained 10 mM phosphate buffer (pH7.4), 300 mM NaCl, 0.1 mg/mL hamster recombinant PrP 90–231 (purified as described in ref. 61), 10 μM thioflavin T (ThT), and 1mM ethylenediaminetetraacetic acid tetrasodium salt (EDTA). Reaction mixes for culture media seeds (from the initial inoculum and samples collected throughout incubation) contained an additional 0.002% SDS in the reaction mix. Organoids were homogenized by motorized pestle to 10% (w/v) in PBS and cleared with a 2000*g* 2 minutes centrifugation. Organoid homogenates were serially diluted in 0.1% SDS/PBS/N2 solution for a final SDS concentration of 0.002% in the reaction mix. For media-seeded and organoid-seeded reactions, respectively, either 80 or 98 μL of reaction mix was loaded into a black 96-well plate with a clear bottom (Nunc), and reaction mixtures were seeded with 20 μL of media or 2 μL of the indicated dilution of organoid homogenate for a final reaction volume of 100 μL and the same final concentrations in the reaction mix as indicated above. Plates were sealed (Nalgene Nunc International sealer) and incubated in a BMG FLUOstar Omega plate reader at 50°C for 50 to 120 hours with cycles of 60 seconds of shaking (700 rpm, double-orbital) and 60 seconds of rest throughout the incubation. ThT fluorescence measurements (excitation, 450 ± 10 nm; emission, 480 ± 10 nm [bottom read]) were taken every 45 minutes. The baseline subtracted maximum value of each well was determined by subtracting the average ThT fluorescence values from the first 3 readings (~2 hours) from the maximum ThT fluorescence value of the well prior to the reaction time cut off. The threshold for a reaction well to be considered positive by RT-QuIC was defined as 10% of the maximum baseline subtracted value on the reaction plate prior to the time cut off. Spearman-Kärber analyses was used to provide estimates of the concentrations of seeding activity units giving positive reactions in 50% of replicate reactions, i.e., the 50% “seeding doses” or SD50’s as previously described (7, 61).

### PK digests and Western blotting.

10% organoid homogenates (wet weight/volume) were treated with 5 μg/mL Proteinase K in 1% Sarkosyl for 1 hour at 37°C with 400 rpm shaking. The reactions were stopped by incubation with 1 μM Pefabloc for 5 minutes at 4°C. Samples were then mixed 1:1 with 2X Bolt LDS sample buffer (Invitrogen) containing 8% β-mercaptoethanol and boiled for 10 minutes. Samples were run on Bolt 4–12% Bis-Tris gels (Invitrogen) and transferred to PVDF membranes using the iBlot 2 transfer system (Invitrogen). Primary antibodies, included 3F4 for PrP (1:10,000; Millipore; MAB1562), NF-L (1:2000; Invitrogen; 13-0400), GFAP (1:10,000; Abcam; ab7260; 1:10K dilution), PSD95 (1:5000; Abcam; ab18258), TOMM20 (1:5000; Abcam; ab186735), LC3B (1:3000; Abcam; ab192890), p62 (1:2000; Abcam; ab109012), Total EGFR (1:1000; Cell Signaling; 2232), and pEGFR (1:1000; Cell Signaling; 2235S). With appropriate species of horseradish peroxidase-conjugated secondary antibodies (Goat anti-Rabbit IgG H&L [HRP; Abcam ab6721] & Goat Anti-Mouse IgG H&L [HRP; abcam ab6789]), markers of interest were visualized using ECL Select (Amersham) and imaged on the iBright imaging system (Invitrogen), which also quantified the band densitometry. With the exception of PK-digested PrP, all band quantifications were normalized to Coomassie staining for total protein.

### Histochemistry.

Organoids were fixed in neutral buffered formalin before paraffin embedding. Five-micron sections underwent deparaffinization, antigen retrieval and staining using the Discovery Ultra-Staining Module. Slides were stained using anti-prion monoclonal antibody 6H4 (1:10000; Prionics) or SAF32 (1:2000; Cayman Chemical; #189720), astrocyte marker polyclonal rabbit anti-glial fibrillary acidic protein (anti-GFAP, 1:3500; Dako; z0334), neuronal markers microtubule associate protein-2 (Map2; 1:2500; Abcam; AB254264) and neurofilament light chain (NFL; 1:500; Invitrogen; 13-0400), and oligodendrocyte marker Olig1 (1:500; Abcam; ab109186) with appropriate secondaries. All histopathology slides were analyzed by observers blinded to the inoculation groups using Aperio Imagescope software.

### Immunofluorescence and confocal imaging.

Five-micron organoid sections underwent deparaffinization and antigen retrieval using the Discovery Ultra-Staining Module. Sections were then blocked in blocking solution (containing 5% (w/v) BSA, 300mM Glycine and 0.1% Triton X-100) for 1 hour at room temperature and labeled with TOMM20 antibody (1:200 dilution; Abcam; ab186735) for overnight at 4°C, washed 3 times with 1×PBS, and labeled with goat anti-rabbit Alexa Fluor 488 secondary antibody (1:500 dilution; Abcam; ab186735) for 1 hour at room temperature. Sections were washed 3 times with 1×PBS, labeled with DAPI (1:10K dilution; ThermoFisher), washed 3 times with 1×PBS, and mounted with ProLong Gold Antifade Mountant (ThermoFisher; P36934). After mounting media were set, the slide surfaces were decontaminated with bleach before imaging with a confocal microscope (Nikon ECLIPSE Ti2). Images were taken with a 40× air objective at Nyquist and processed by Huygens Professional (25.04), including deconvolution and rendering.

### PrestoBlue assay.

PrestoBlue metabolism was measured as per the manufacturer’s instructions. Briefly, PrestoBlue reagent (Thermo Fisher Scientific) was diluted 1 in 10 in organoid media. Existing organoid media was removed and organoids were incubated in Prestoblue-containing media for 30 minutes. The metabolized Prestoblue-containing media was then transferred into replicate wells for analysis. PrestoBlue fluorescence was measured at 560 nm excitation and 590 nm emission in a ClarioStar plate reader (BMG).

### BioPlex signaling panels.

BioPlex cell signaling panels MAPK 9-plex (catalogue # LQ00000S6KL81S) and AKT 8-plex (catalogue # LQ00006JK0K0RR) panels were run according to BioPlex instructions using the BioPlex 3D suspension array system. Briefly, organoids were triturated in 200 μL cell lysis buffer including PMSF and factor QG (BioRad). Samples were then analyzed using a BCA assay and further diluted in cell lysis buffer to ensure all samples were in a range appropriate for the BioPlex cell signaling assays. Mean fluorescence readings for each analyte were normalized to total protein as determined by BCA and are shown plotted relative to the NBH control. Complete data can be found in Supplemental Table 4.

### Electrophysiology.

The neuroelectrophysiology, based on extracellular neuronal activity, was measured using 24-well multielectrode arrays (Multiwell-MEA; Multichannel systems) with 12 electrodes per well. Cerebral organoids were embedded onto the electrodes by Matrigel Matrix (Corning; Supplemental Figure 5) or laminin (Corning), as described in ref. 62. The embedded organoids were incubated unmoved for a few days to allow strong adherence to the electrodes before recording their electrophysiology and remained in situ throughout the experiment. The local field potential (LFP) sampling at 20 KHz was recorded for 5 minutes at 90 and 180 dpi using a Multiwell Screen software (version 1.11.6.0; Multi-Channel Systems). A Multiwell Analyzer software (version 1.8.5.0; Multi-Channel Systems) was used to filter the LFP with a 300–3500 Hz high-pass second-order Butterworth filter and extract neuronal population spikes. Due to higher noise-to-signal ratio in organoids plated with Matrigel Matrix, neuronal population spiking was detected by a slope detection method with a window of 3,000 μs, a minimum-maximum rise time of 100–1,000 μs, and an amplitude threshold of 3 standard deviations above the mean amplitude of the noise (measured within the first 1,000 ms). A threshold detection method, with 4 standard deviations above the mean amplitude, was used to detect spikes in organoids plated with Laminin. Due to a substantial level of low frequency spindles detected in organoids plated with Laminin, the filter was changed to a low-pass filter < 1000 Hz to account for any phase-coupling spiking. The neuronal burst was detected as a minimum of 4 spikes firing less than 50 ms apart for a duration of at least 50 ms. The minimal interval between bursts was 100 ms. Neurophysiological parameters including spike rate, burst rate, and peak-to-peak amplitudes were extracted for further statistical analyses.

### RNA extraction and purification.

RNA was extracted from 180–200 dpi organoids using the TRIzol Reagent extraction method via manufacturer protocol (Invitrogen catalog #15596026). Contaminating genomic DNA was reduced by adding 2 Units of DNase I (RNase-free) and the appropriate amount of 10X DNase buffer (Ambion) to each sample then incubated for 1 hour at 23°C. Final RNA Purification was then carried out by RNA Clean & Concentrator from Zymo Research (catalog #R1017).

### Quantitative Real-Time PCR (qRT-PCR) and analysis.

Four-hundred ng of RNA from each sample was reverse transcribed to synthesize cDNA using the RT2 First Stand Kit per manufacturer’s instructions (Qiagen). Each cDNA reaction was mixed with 2× RT^2^ SYBR Green qPCR Mastermix purchased from Qiagen with RNase-free water to a final volume of 1.3 mL. Ten microliters of the mixture was then added to each well of a 384-well format plate of the Human Neurotransmitter Receptor Array PAHS-060ZE (Qiagen), Human Mitochondria Array PAHS-087Z (Qiagen) or Human EGF / PDGF Signaling Pathway PAHS-040ZE (Qiagen).

The assay was carried out on an Applied Biosystems ViiA 7 Real-Time PCR System with a 384-well block using the following conditions: 1 cycle at 10 minutes, 95°C; 40 cycles at 15 seconds, 95°C then 1 minute, 60°C with fluorescence data collection. Melting curves were generated at the end of the completed run to determine the quality of the reaction products. Raw threshold cycle (CT) data was collected with a CT of 35 as the cutoff. CT data was analyzed using the web-based RT^2^ Profiler PCR Array Data Analysis from Qiagen. All CT values were normalized to the geometric mean of the CT values for the housekeeping genes ACTB and GAPDH. Changes in transcription were calculated by the software using the ΔΔCT based method. A mean of ≥ or ≤ 2.0-fold change and *P* value of ≤ 0.05 considered significant. For qRT-PCR data the *P* values were not adjusted for multiple comparisons since we were interested in only controlling for the individual error rate, where an adjustment for multiple tests is deemed unnecessary. Results are shown as fold change from the NBH control of the respective batch. Complete datasets can be found in Supplemental Table 1.

### Mitochondrial membrane potential assay.

Mitochondrial membrane potential was determined using a JC-1 fluorescence-based mitochondrial membrane potential assay kit (Abcam) per the manufacturers’ instructions.

### Complex 1 activity assay.

The mitochondrial complex 1 activity was measured using the Complex 1 Enzyme Microplate Assay Kit (Colorimetric; Abcam) as described in Foliaki et al. (63). Briefly, organoids were lysed in detergent, and the protein concentration was measured by BCA assay and corrected across samples. The lysates were added to the microplate (200 μL/Sample/well) and incubated at room temperature for 3 hours. Wells were washed 3 times with 1× Wash buffer and then filled with the assay solution (200 μL/well), which contained 1× Dilution buffer, 1× NADH, and 1× Dye. The complex 1 activity was measured in a ClarioStar plate reader (BMG) at 450 nm wavelength for 120 minutes (at room temperature) in 30-second intervals with a shake between readings.

### 10X single cell sequencing.

Organoids (infected and batch-matched NBH controls) were gently dissociated by washing in sterile PBS, followed by digestion in pre-warmed 50:50 Accutase:Trypsin solution (Sigma Aldrich) for 30 minutes at 37°C with mixing at 65 rpm. At the start of the digestion organoids were triturated through a wide-bore P1000 pipette tip 5 times and this was repeated 5 minutes before the end of the incubation. At the end of the incubation, the cell suspension was gently triturated through a standard P1000 tip to break up any cells clumps and dislodge any cells that were loosely still attached to the organoid exterior. Care was taken not to aspirate the remaining organoid tissue to avoid including any potentially necrotic cells from the organoid core. The cell suspension was filtered through a 40 μm sterile filter into an equal volume of 2× ice cold stop solution (20% [v/v] FBS in PBS), then kept on ice or at 4°C for the remaining steps of the procedure. Cells were counted using trypan blue and a hemocytometer, centrifuged at 300*g* for 5 minutes, and resuspended in an appropriate amount of assay buffer (sterile PBS with 0.5% w/v BSA). A sufficient volume for a target quantity of 8,000 cells was added to each reaction. 10X cell separation, reverse-transcription, barcoding and amplification were carried out according to the manufacturer’s instructions. Sequencing was performed at the Research Technologies Branch of NIAID using a Nextseq 2000 sequencer. 10X SCS data has been deposited in the NCBI GEO database under identifier GSE309815 (www.ncbi.nlm.nih.gov/geo/query/acc.cgi?acc=GSE309815).

Single-cell gene expression data were processed by the Research Technologies Branch of NIAID using the Seurat framework (64). Cells with fewer than 300 detected genes or more than 15% mitochondrial gene content were excluded from downstream analyses; no additional filtering based on read depth was applied. To account for variability across pooled organoids, canonical correlation analysis–based (CCA-based) integration (65) was performed. Data were normalized using log-normalization, scaled, and subjected to clustering using the Louvain algorithm with a resolution parameter of 0.2. Differential gene expression analysis was carried out using the MAST statistical framework (66), applying an adjusted *P* value threshold of < 0.05 and a log_2_ fold-change cutoff of |0.25| to define significantly differentially expressed genes (DEGs; Supplemental Table 2). The overall workflow followed the publicly available cell-seek pipeline (https://github.com/OpenOmics/cell-seek) (Commit ID: Commit f970551).

Cell type identification was performed manually using the upregulated DEGs from each cluster cross-referenced with CellMarker 2.0 (67) and The Human Protein Atlas (68) databases. A heatmap of gene expression associated with different cell types is shown with the cell type identifications in Supplemental Figure 8. DEGs for each of the cell types for each infection versus NBH controls were entered into Ingenuity Pathway Analysis (IPA: Qiagen). IPA core analysis was performed using the DEGs and a stringent match with human gene pathways. All cell types were compared using comparison analysis with a cut off –log(B-H *P*-value) of 2. Raw and graphed data shown in these analyses are in Supplemental Table 3 and Supplemental Figure 11).

### Statistics.

Statistical analysis, including Mann Whitney *U* test (2-tailed), Student’s *t* test (2-tailed), 1-way ANOVA with Tukey’s secondary test, 1-way ANOVA with Dunnett’s secondary test, and 2-way ANOVA with Dunnett’s secondary test, are indicated in figure legends and were carried out in GraphPad Prism 7.04. Unless otherwise stated, *P* < 0.05 was considered significant. The data presented herein can be found in the Supplemental Files as indicated in the relevant method section. All other data are in Supplemental Table 5. Graphs show the mean and standard deviation unless otherwise indicated.

### Study approval.

KYOU-DXR0109B human iPSCs were obtained from the American Type Culture Collection with no donor identifying information. Similarly, all brain tissues used in this study were obtained on autopsy and provided de-identified. Thus, the NIH Office of Human Subjects Research Protections (OHSRP) has deemed these samples exempt from IRB review.

### Data availability.

All data applicable to this manuscript are available within the manuscript and its attached supplemental materials, and values for all data points in graphs are reported in the Supporting Data Values file. Raw single cell sequencing files have been deposited in the NCBI gene expression omnibus under ID GSE309815.

## Author contributions

KW, BRG, ROW, and CLH conceived the project and designed the work. KW, BRG, STF, BR, AH, ROW, TT, JAC, and CLH acquired and/or analyzed data. KW, BRG, ROW, GZ, and CLH interpreted the data. KW, BRG, GZ, JAC, and CLH drafted and/or substantially revised the manuscript. All authors have approved the submitted version and agree to be personally accountable for their work. Co-authorship of the two first authors was assigned based on equal input, both in scientific activity and intellectual contribution, to the final manuscript.

## Conflict of interest

The authors have declared that no conflict of interest exists.

## Funding support

This work is the result of NIH funding, in whole or in part, and is subject to the NIH Public Access Policy. Through acceptance of this federal funding, the NIH has been given a right to make the work publicly available in PubMed Central.

- The Intramural Research Program of the National Institutes of Health (NIH).
- CJD Foundation research grant (to CLH).
- CJF Foundation Fellowship (to AH).

## Supplementary Material

Supplemental data

Unedited blot and gel images

Supplemental table 1

Supplemental table 2

Supplemental table 3

Supplemental table 4

Supplemental table 5

Supporting data values

## Figures and Tables

**Table 2:**
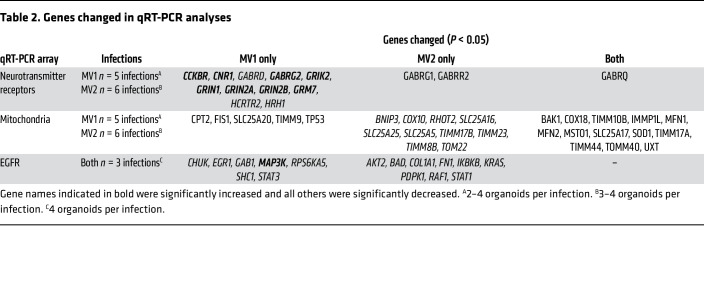
Genes changed in qRT-PCR analyses

**Table 1:**
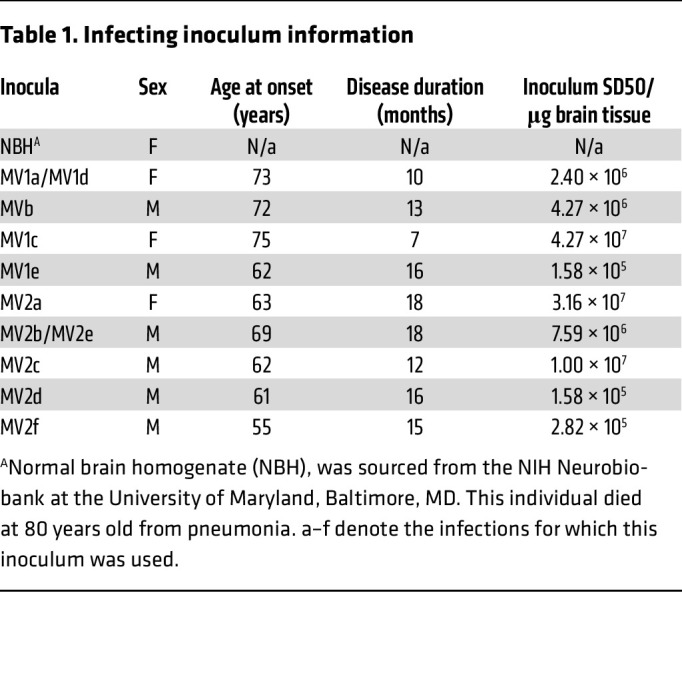
Infecting inoculum information

## Version 1 02/12/2026

In-Press Preview

## Version 2 04/15/2026

Electronic publication
