# Supplementary material for: Distinct neuronal alterations distinguish two subtypes of sporadic Creutzfeldt-Jakob disease with shared dysfunctional pathways
Source: J Clin Invest. 2026 Feb 12;136(8):e194721. doi: 10.1172/JCI194721 (PMC13078861; doi:10.1172/JCI194721)

Katie Williams, Bradley R. Groveman, Simote T. Foliaki, Brent Race, Arielle Hay, Ryan O. Walters, Tina Thomas, Gianluigi Zanusso, James A. Carroll, Cathryn L. Haigh. **Two sporadic Creutzfeldt-Jakob Disease subtypes demonstrate common dysfunctional pathways with differing characteristics in vitro.**

Supplemental Figure 1*. Further PrP blots and IHC.* **A.** Western blots of protease resistant PrP (detected with 3F4 antibody) in 3 (1-3) infected organoids at 90 and 175dpi. **B.** PrP (6H4; brown pigment) IHC staining examples from organoid infections.

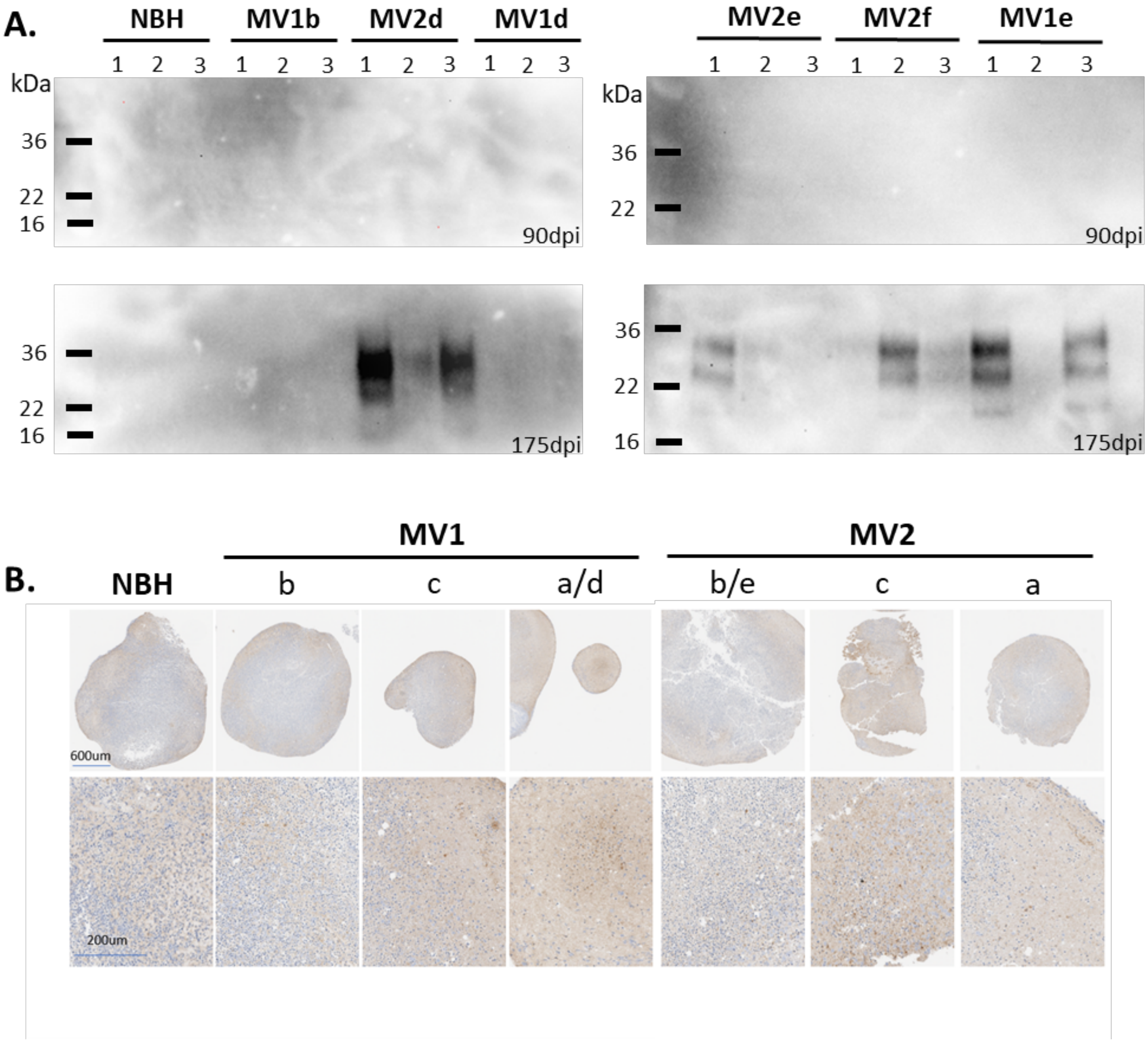

Katie Williams, Bradley R. Groveman, Simote T. Foliaki, Brent Race, Arielle Hay, Ryan O. Walters, Tina Thomas, Gianluigi Zanusso, James A. Carroll, Cathryn L. Haigh. **Two sporadic Creutzfeldt-Jakob Disease subtypes demonstrate common dysfunctional pathways with differing characteristics in vitro.**

Supplemental Figure 2. *Astrogliosis.* **A.** Western blots of GFAP detection in infected organoids. Each lane shows a single organoid. **B.** Densitometric quantification of A at 180 dpi. **C.** Example GFAP detection in organoids by IHC. Infections with the highest GFAP detection did not correlate with those that showed the greatest PrP deposition. One-way ANOVA with Welch's correction, *p<0.05, **p<0.01, ***P<0.001.

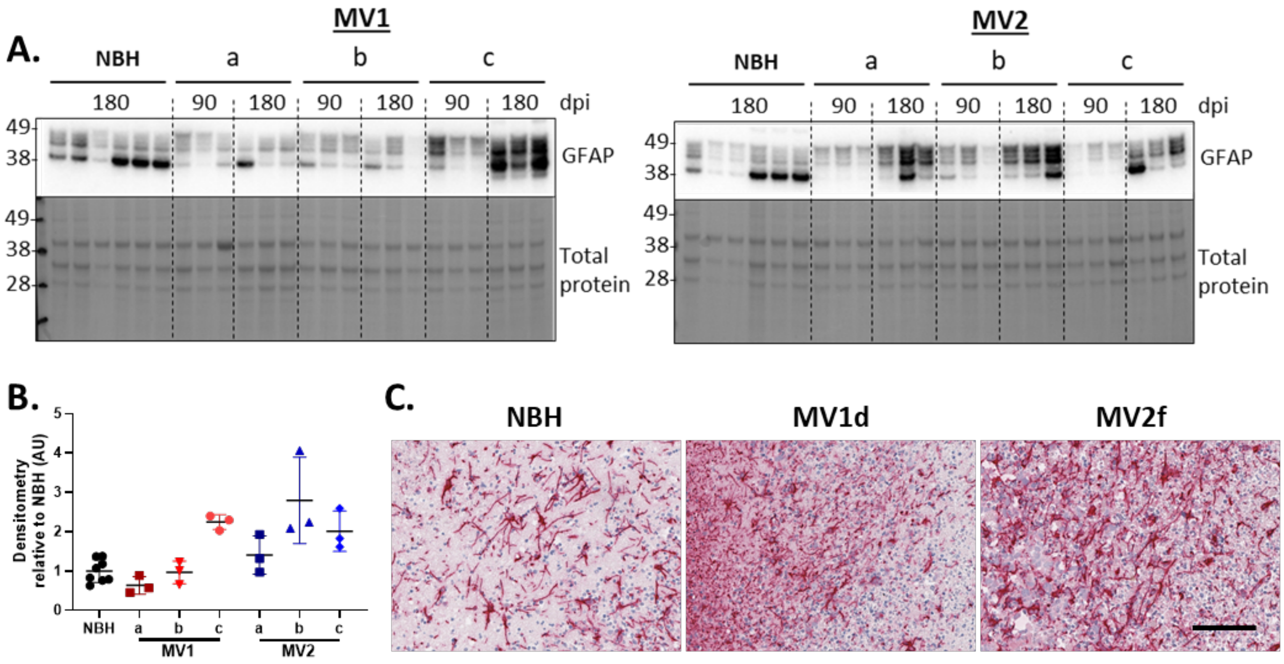

Katie Williams, Bradley R. Groveman, Simote T. Foliaki, Brent Race, Arielle Hay, Ryan O. Walters, Tina Thomas, Gianluigi Zanusso, James A. Carroll, Cathryn L. Haigh. **Two sporadic Creutzfeldt-Jakob Disease subtypes demonstrate common dysfunctional pathways with differing characteristics in vitro.**

Supplemental Figure 3. *H&E staining.* Examples of spongiform change in H&E stained organoids from NBH and two each of MV1 and MV2 infections. Although some vacuolation is observed in the infections, some is also present in the controls indicating a background level in the organoids that makes it difficult to discern what are disease-related changes.

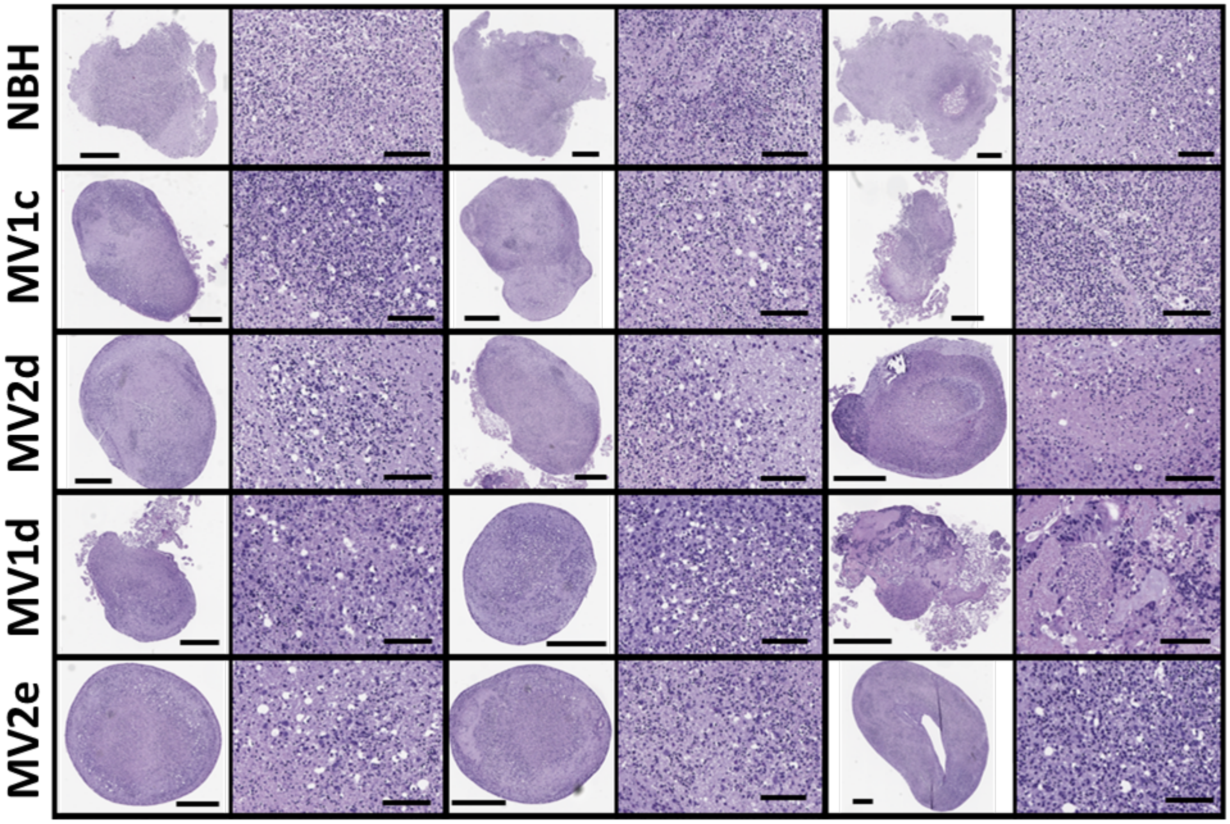

Katie Williams, Bradley R. Groveman, Simote T. Foliaki, Brent Race, Arielle Hay, Ryan O. Walters, Tina Thomas, Gianluigi Zanusso, James A. Carroll, Cathryn L. Haigh. **Two sporadic Creutzfeldt-Jakob Disease subtypes demonstrate common dysfunctional pathways with differing characteristics in vitro.**

Supplemental Figure 4. *Cell death pathways are not activated in the organoids up to 180 dpi.* **A.** Organoids were monitored for changes in their viability by prestoblue viability assay at 175-180 dpi and did not show any reduction in prestoblue metabolism. **B.** The phospho-protein levels of three each of the MV1 and MV2 infections were examined for p53, BAD and mTOR, which are involved in apoptosis and autophagy pathways. Bioplex detection at 90 (n = 3) and 180 (n = 6) dpi relative to the respective time point NBH control (indicated by the dotted line). Shown are the mean and standard deviation of three infections per subtype, lines/colors indicate the same infection sampled at each time point. Few statistically significant changes were seen but mostly these were in the direction of decreased phospho-protein levels, which would indicate less activation. **C.** The unchanged or decreased levels of proteins associated with cell death pathways were confirm by qRT-PCR analysis of several target genes, shown as fold change from the NBH control (indicated by the dotted line; NBH n = 3 mock infections with 3-4 organoids sampled for each). Some analyses were done on all infections and some only on the same infections as used for phospho-protein analysis. For analyses with all infections, each dot is an individual infection, and the shading of the dot indicates the number of organoids sampled and averaged for that infection, and for the analyses with just three infections, each dot is an individual infection for which 4 organoids were sampled and averaged. The gene transcript changes supported that there was no increase in cell death associated transcripts. *$p<0.05$, **$p<0.01$, ***$p<0.001$, 1-way ANOVA (A), 2-way ANOVA (B, colored asterisks indicate the significant infections), students t-test (C).

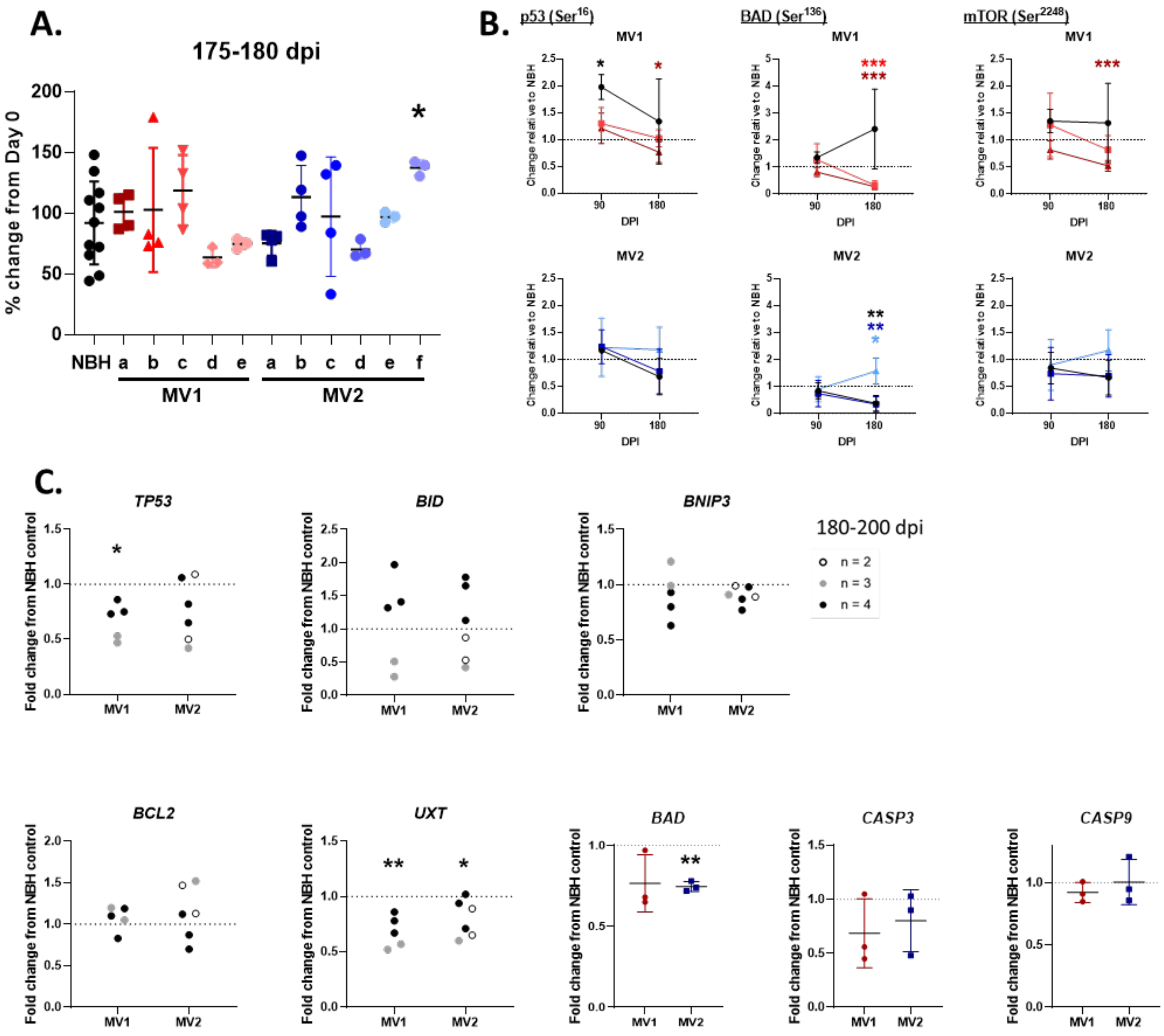
A.
175-180 dpi
% change from Day 0
NBH a b c d e a b c d e f
MV1
MV2
B.
p53 (Ser16)
BAD (Ser136)
mTOR (Ser2248)
MV1
MV2
Change relative to NBH
DPI
C.
TP53
BID
BNIP3
BCL2
UXT
BAD
CASP3
CASP9
Fold change from NBH control
180-200 dpi
n = 2
n = 3
n = 4

Katie Williams, Bradley R. Groveman, Simote T. Foliaki, Brent Race, Arielle Hay, Ryan O. Walters, Tina Thomas, Gianluigi Zanusso, James A. Carroll, Cathryn L. Haigh. **Two sporadic Creutzfeldt-Jakob Disease subtypes demonstrate common dysfunctional pathways with differing characteristics in vitro.**

Supplemental Figure 5. *Further electrophysiology results.* **A & B.** Individual spike (A) and burst (B) results for each of the infections shown amalgamated in Figure 2C and D. **C & D.** Organoids from infections d-f were plated in 24-well MEA plates at ~90 dpi and allowed to adhere to the electrodes. Unlike those in A & B, these organoids remained in situ for the duration of the infection, thus the same organoids were monitored at both time points. However, the long duration of culture within the plate caused loss of some organoid architecture. Graphs show combined and individual spike (C) and burst (D) rates at 100 and 200 dpi. Each dot is an individual organoid. Analysis was done by one-way ANOVA with welches correction where standard deviations were not equal; **p<0.01, ***p<0.001, ****p<0.0001.

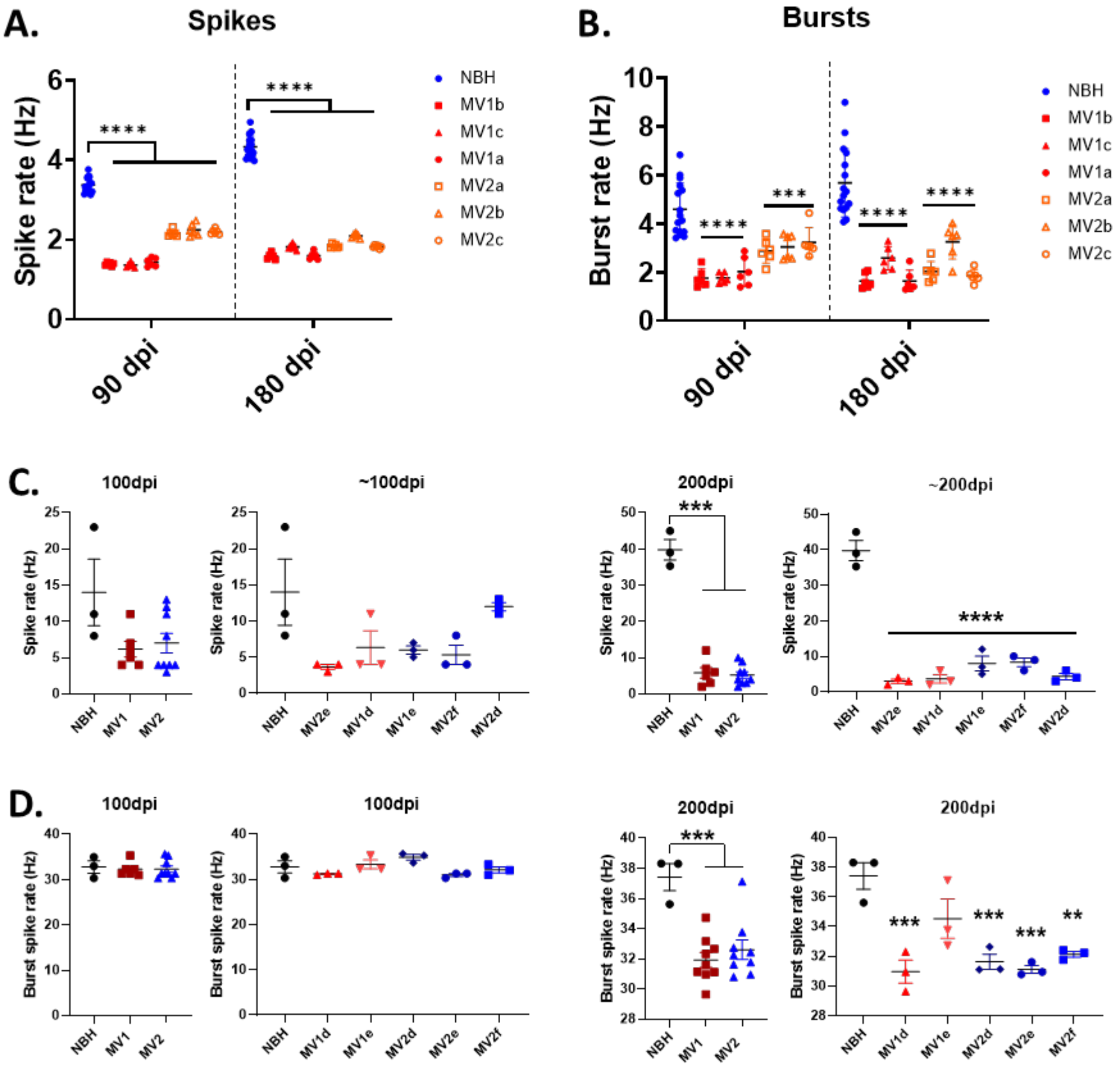

Katie Williams, Bradley R. Groveman, Simote T. Foliaki, Brent Race, Arielle Hay, Ryan O. Walters, Tina Thomas, Gianluigi Zanusso, James A. Carroll, Cathryn L. Haigh. **Two sporadic Creutzfeldt-Jakob Disease subtypes demonstrate common dysfunctional pathways with differing characteristics in vitro.**

Supplemental Figure 6*. Correlations between seeding activity or PrP deposition and other measured parameters.* **A.** Correlations of seeding activity with electrophysiological parameters. Both readings were taken from the same organoids; each point is a single organoid. **B.** Correlations of western blot densitometry quantification of protease resistant PrP with other proteins probed by western blotting at 180 dpi. Samples were taken from the same organoids; each point is a single organoid. Linear regression analysis was performed on the MV1 and MV2 infections separately and after combination of all infected organoids. No significant changes were found for the MV1 or MV2 infections; however, two significantly non-linear correlations were identified when all samples were combined and these are shown with the respective p-value.

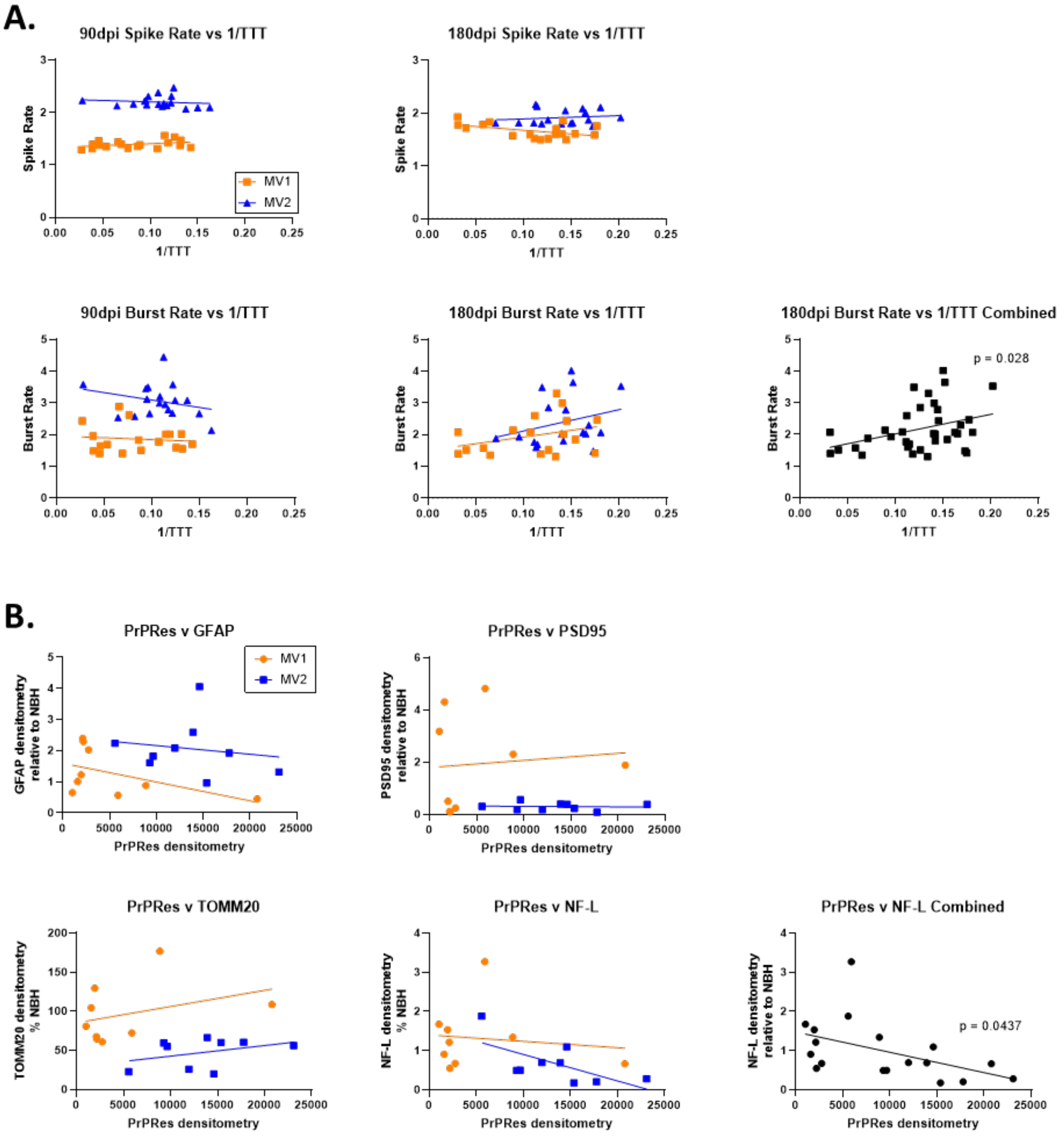

Supplemental Figure 7. *Further signaling pathway intermediate phosphorylation assessments and pEGFR/EGFR western blotting.* **A.** Bioplex analysis of phosphorylated signal transduction intermediates. Three MV1 and MV2 infections were tested. Different colored dots and lines denote 90 (n = 3) and 180 (n = 6) dpi samples (organoids) from the same infection. Colored asterisks show changes that were significant corresponding to each infection *p<0.05, ***p<0.001, 2-way ANOVA with Dunnett's secondary test. NBH controls are indicated by the dotted lines. **B.** Western blotting for pEGFR & EGFR with total protein (Coomassie) loading controls. Please note that the total protein stain is the same as that shown in 3E as multiple probes of the same membrane were performed.

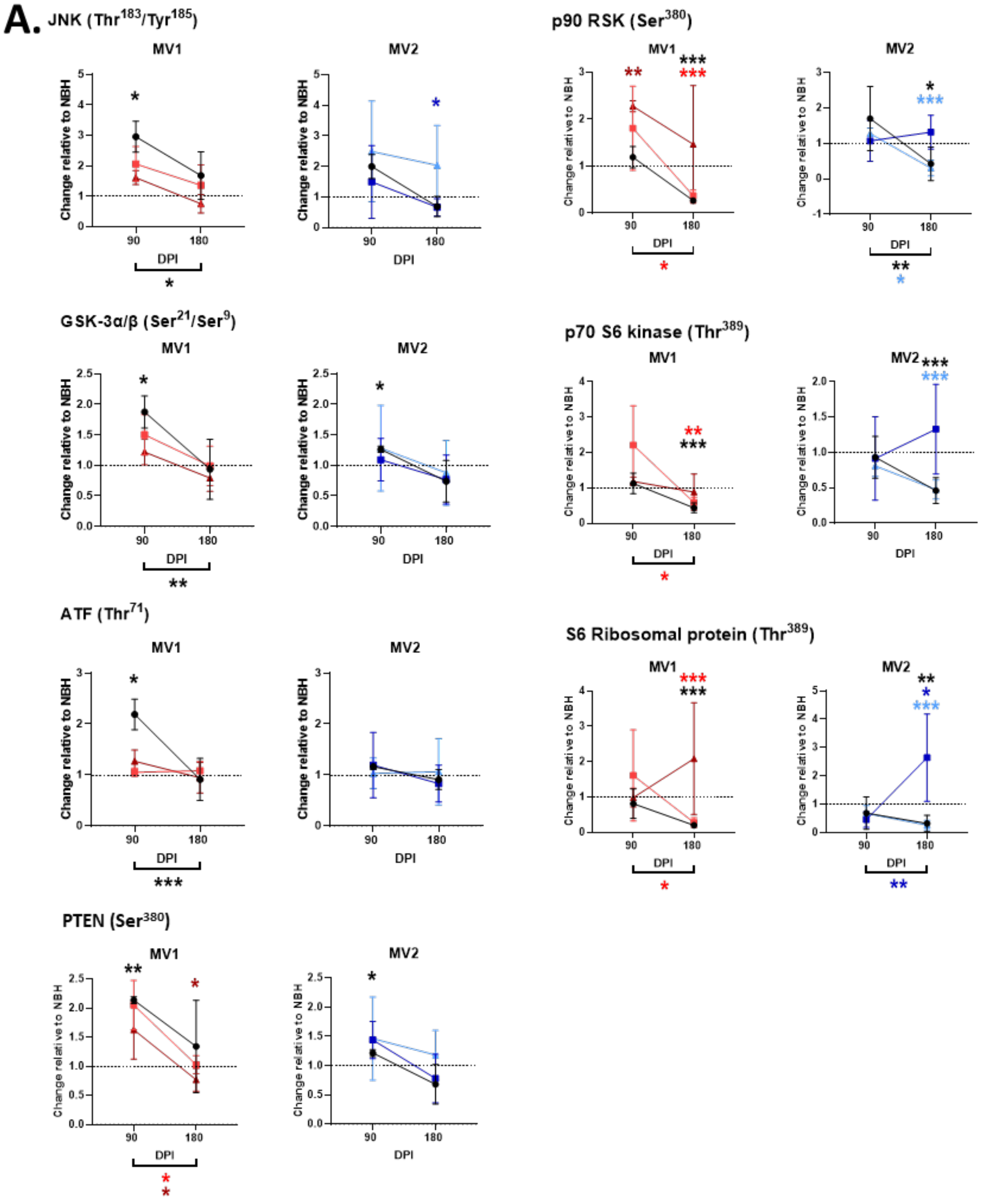

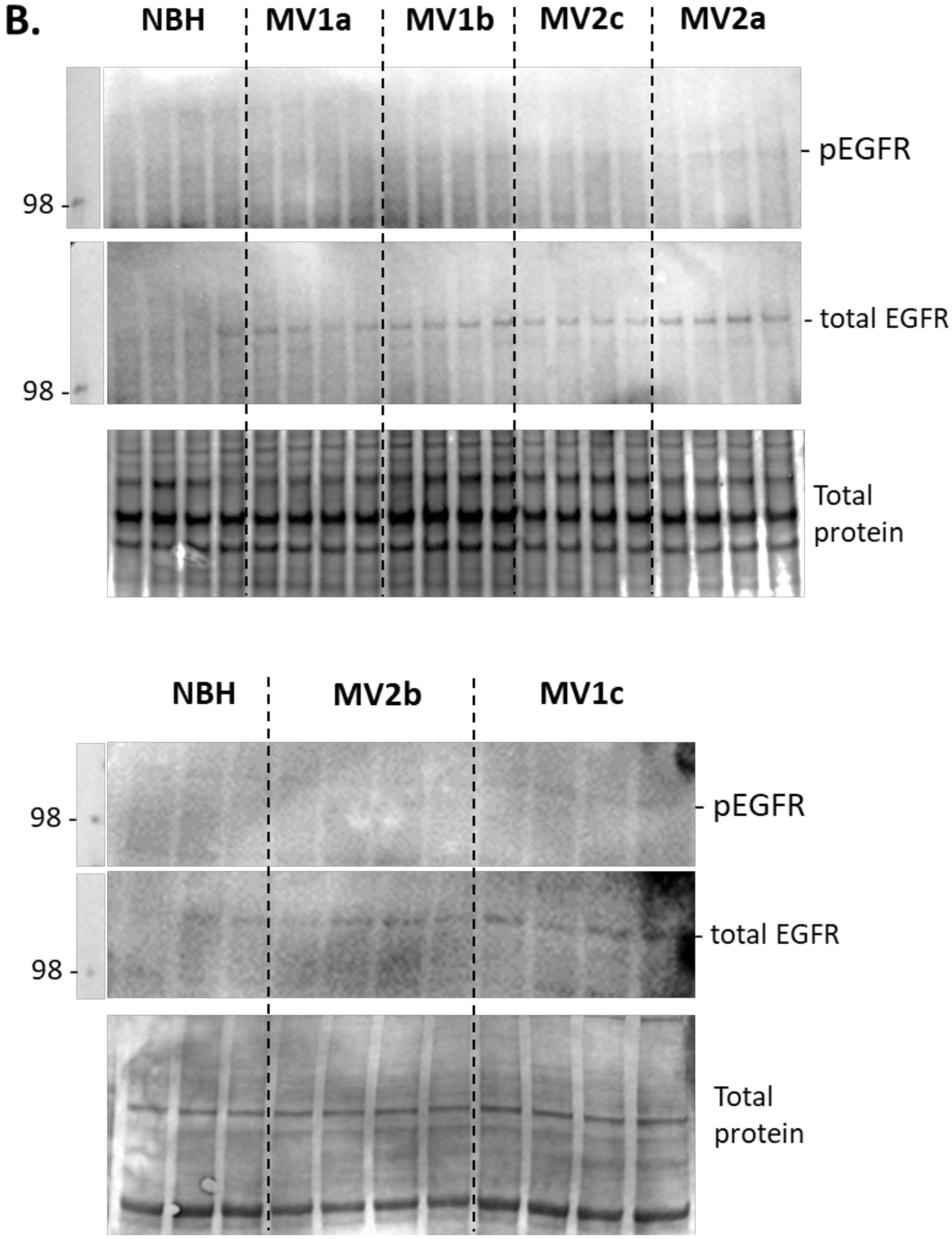
B.
NBH
MV1a
MV1b
MV2c
MV2a
pEGFR
98
total EGFR
98
Total protein
NBH
MV2b
MV1c
98
pEGFR
98
total EGFR
Total protein

Katie Williams, Bradley R. Groveman, Simote T. Foliaki, Brent Race, Arielle Hay, Ryan O. Walters, Tina Thomas, Gianluigi Zanusso, James A. Carroll, Cathryn L. Haigh. **Two sporadic Creutzfeldt-Jakob Disease subtypes demonstrate common dysfunctional pathways with differing characteristics in vitro.**

Supplemental Figure 8*. 10x single cell sequencing infection quantification and lineage identification.* **A & B.** RT-QuIC reaction curves (A) and dilution series quantification from residual cell samples taken from the same organoids as used for the single cell sequencing (MV1d, MV2e), with reaction intensities compared against sCJD brain homogenate. **C.** Analysis of genes enriched in individual clusters for identifying the cluster cell type shown as a heatmap of genes enriched in each cluster grouped by cell lineage identity. **D.** Quality control violin plots of nCounts, nFeatures, and percent mitochondria for each group and each infection compared with NBH controls. **E.** Volcano plots of gene changes for each group and each infection compared with NBH controls. X and y axis dotted lines denote significance cutoffs as defined for the DEGs. Some significantly change genes are annotated.

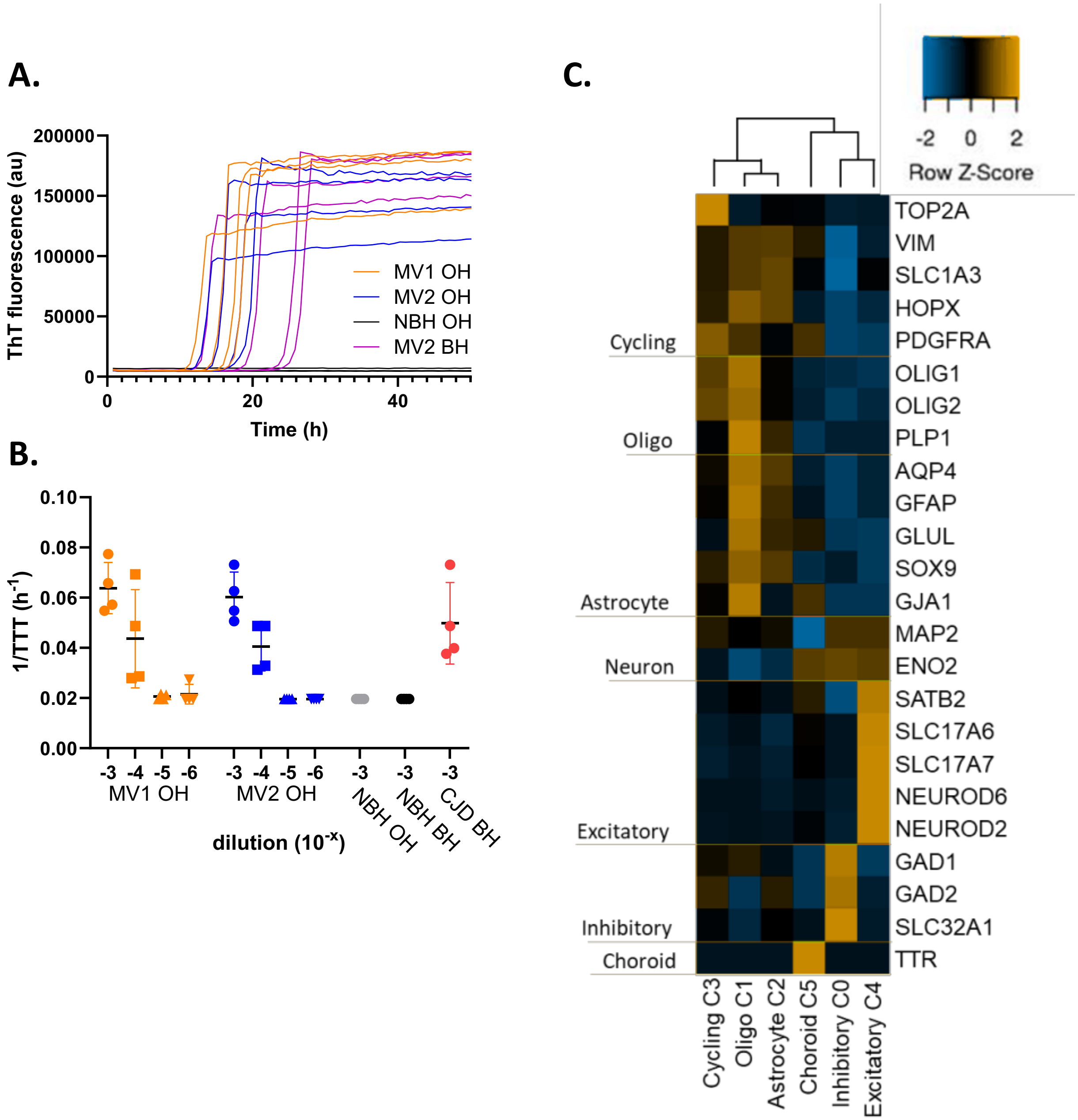

D.

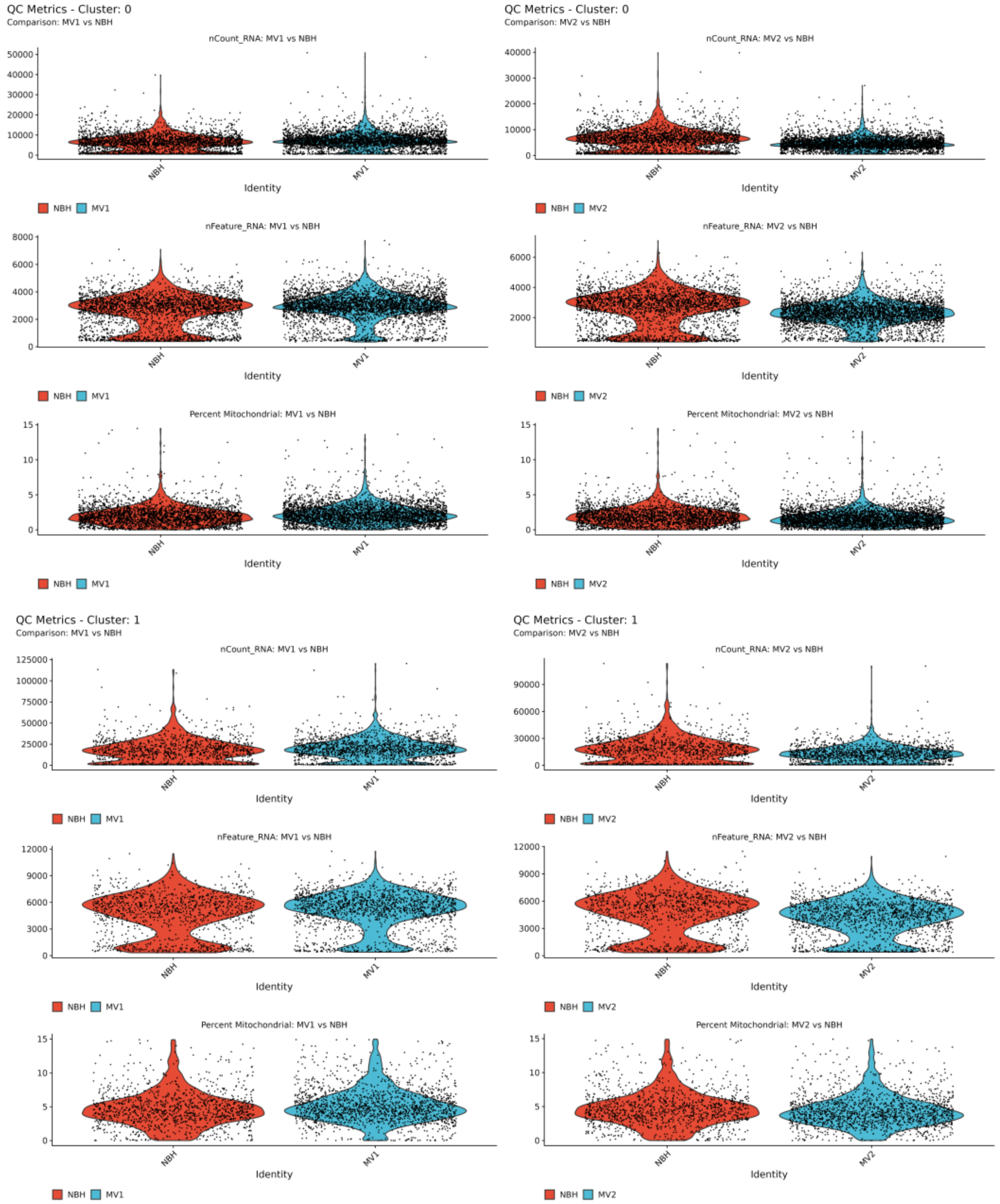
QC Metrics - Cluster: 0
Comparison: MV1 vs NBH
nCount_RNA: MV1 vs NBH
nFeature_RNA: MV1 vs NBH
Percent Mitochondrial: MV1 vs NBH
QC Metrics - Cluster: 0
Comparison: MV2 vs NBH
nCount_RNA: MV2 vs NBH
nFeature_RNA: MV2 vs NBH
Percent Mitochondrial: MV2 vs NBH
QC Metrics - Cluster: 1
Comparison: MV1 vs NBH
QC Metrics - Cluster: 1
Comparison: MV2 vs NBH
Identity
NBH
MV1
MV2

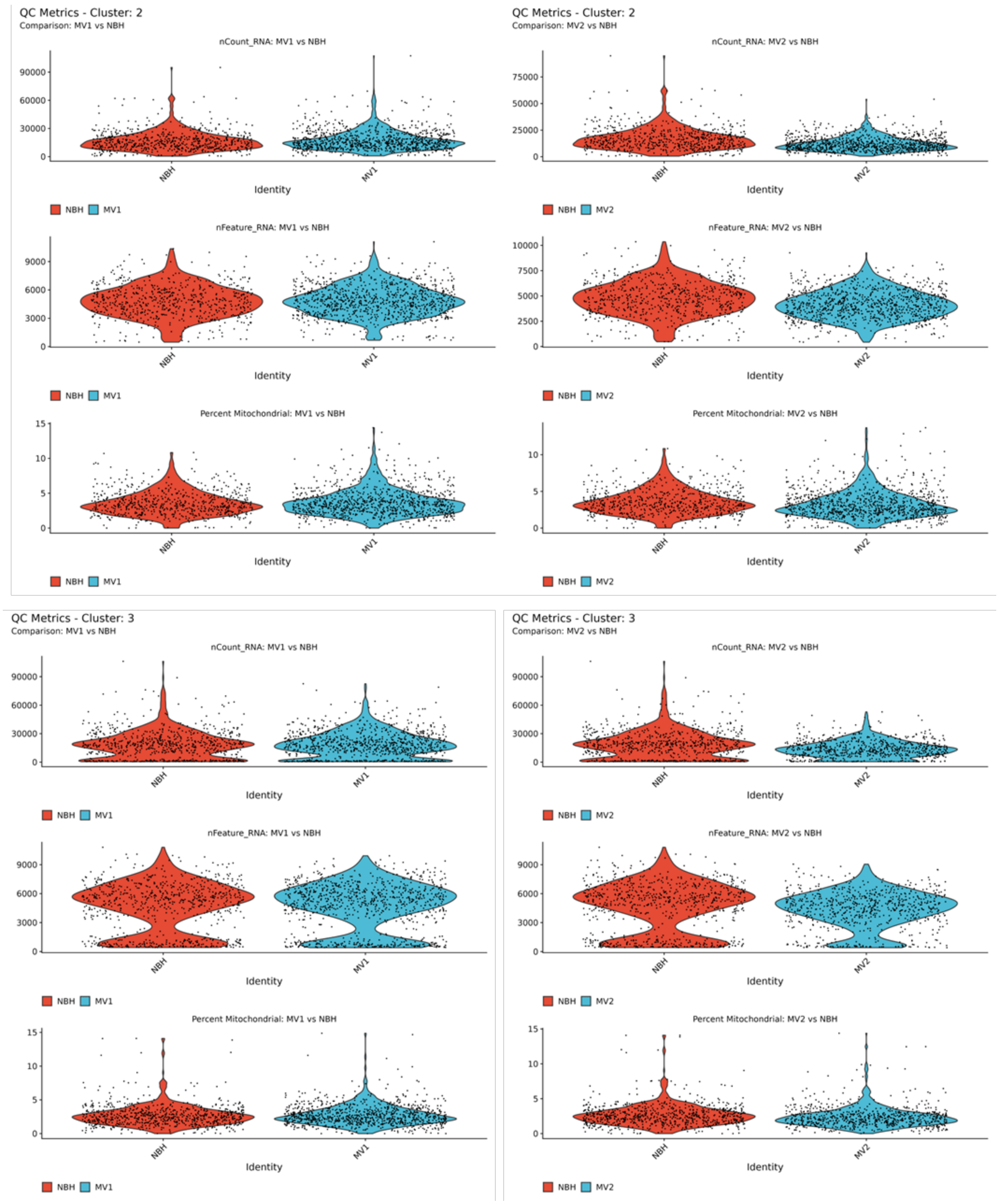
QC Metrics - Cluster: 2
Comparison: MV1 vs NBH
nCount_RNA: MV1 vs NBH
nFeature_RNA: MV1 vs NBH
Percent Mitochondrial: MV1 vs NBH
QC Metrics - Cluster: 2
Comparison: MV2 vs NBH
nCount_RNA: MV2 vs NBH
nFeature_RNA: MV2 vs NBH
Percent Mitochondrial: MV2 vs NBH
QC Metrics - Cluster: 3
Comparison: MV1 vs NBH
nCount_RNA: MV1 vs NBH
nFeature_RNA: MV1 vs NBH
Percent Mitochondrial: MV1 vs NBH
QC Metrics - Cluster: 3
Comparison: MV2 vs NBH
nCount_RNA: MV2 vs NBH
nFeature_RNA: MV2 vs NBH
Percent Mitochondrial: MV2 vs NBH
Identity
NBH
MV1
MV2

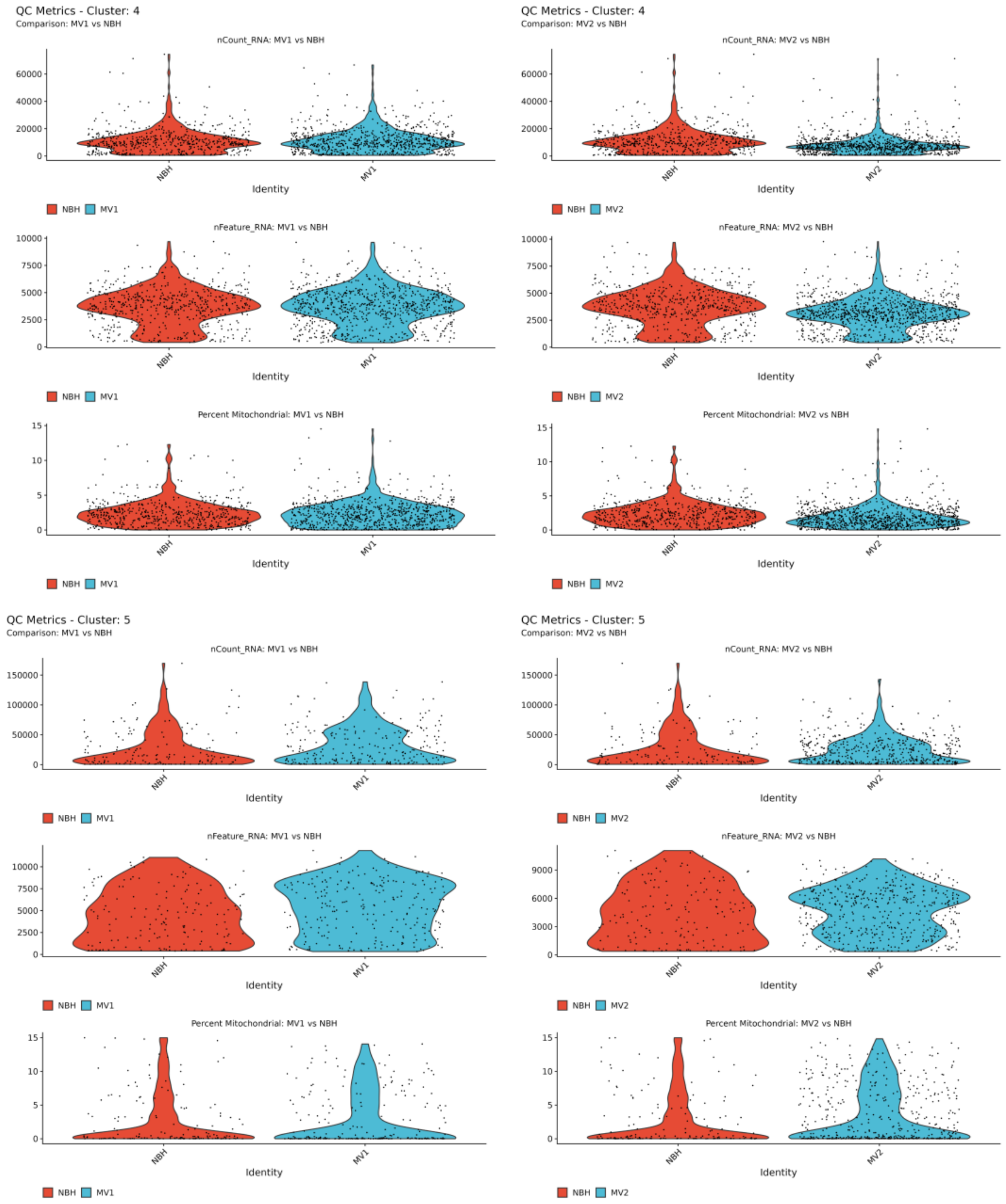
QC Metrics - Cluster: 4
Comparison: MV1 vs NBH
nCount_RNA: MV1 vs NBH
nFeature_RNA: MV1 vs NBH
Percent Mitochondrial: MV1 vs NBH
QC Metrics - Cluster: 4
Comparison: MV2 vs NBH
nCount_RNA: MV2 vs NBH
nFeature_RNA: MV2 vs NBH
Percent Mitochondrial: MV2 vs NBH
QC Metrics - Cluster: 5
Comparison: MV1 vs NBH
nCount_RNA: MV1 vs NBH
nFeature_RNA: MV1 vs NBH
Percent Mitochondrial: MV1 vs NBH
QC Metrics - Cluster: 5
Comparison: MV2 vs NBH
nCount_RNA: MV2 vs NBH
nFeature_RNA: MV2 vs NBH
Percent Mitochondrial: MV2 vs NBH
Identity
NBH
MV1
MV2

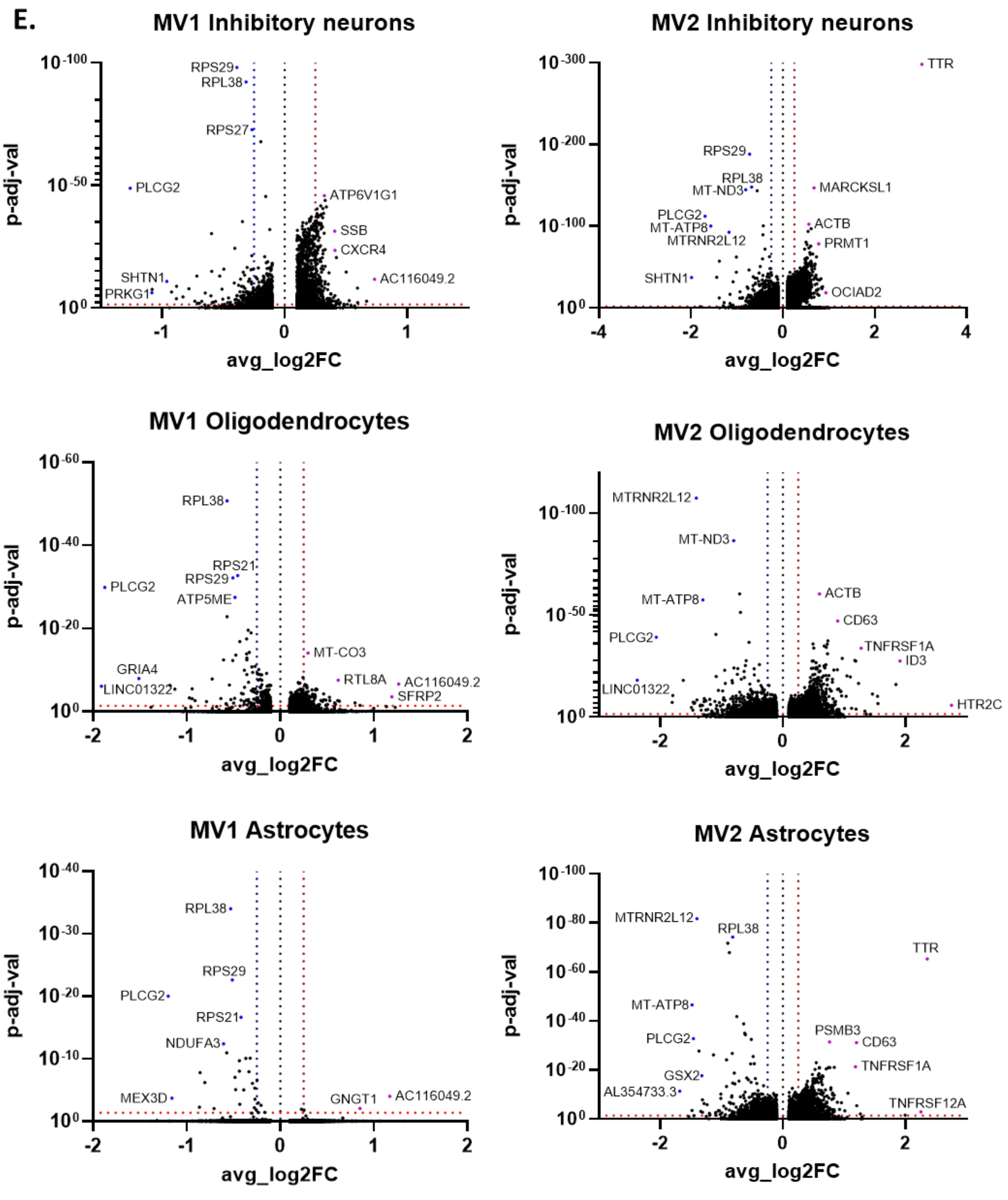
E.
MV1 Inhibitory neurons
MV2 Inhibitory neurons
MV1 Oligodendrocytes
MV2 Oligodendrocytes
MV1 Astrocytes
MV2 Astrocytes
p-adj-val
avg_log2FC
RPS29
RPL38
RPS27
PLCG2
ATP6V1G1
SSB
CXCR4
AC116049.2
SHTN1
PRKG1
TTR
MARCKSL1
MT-ND3
PLCG2
MT-ATP8
MTRNR2L12
ACTB
PRMT1
OCIAD2
RPS21
ATP5ME
MT-CO3
RTL8A
SFRP2
GRIA4
LINC01322
CD63
TNFRSF1A
ID3
HTR2C
NDUFA3
MEX3D
GNGT1
PSMB3
GSX2
AL354733.3
TNFRSF12A

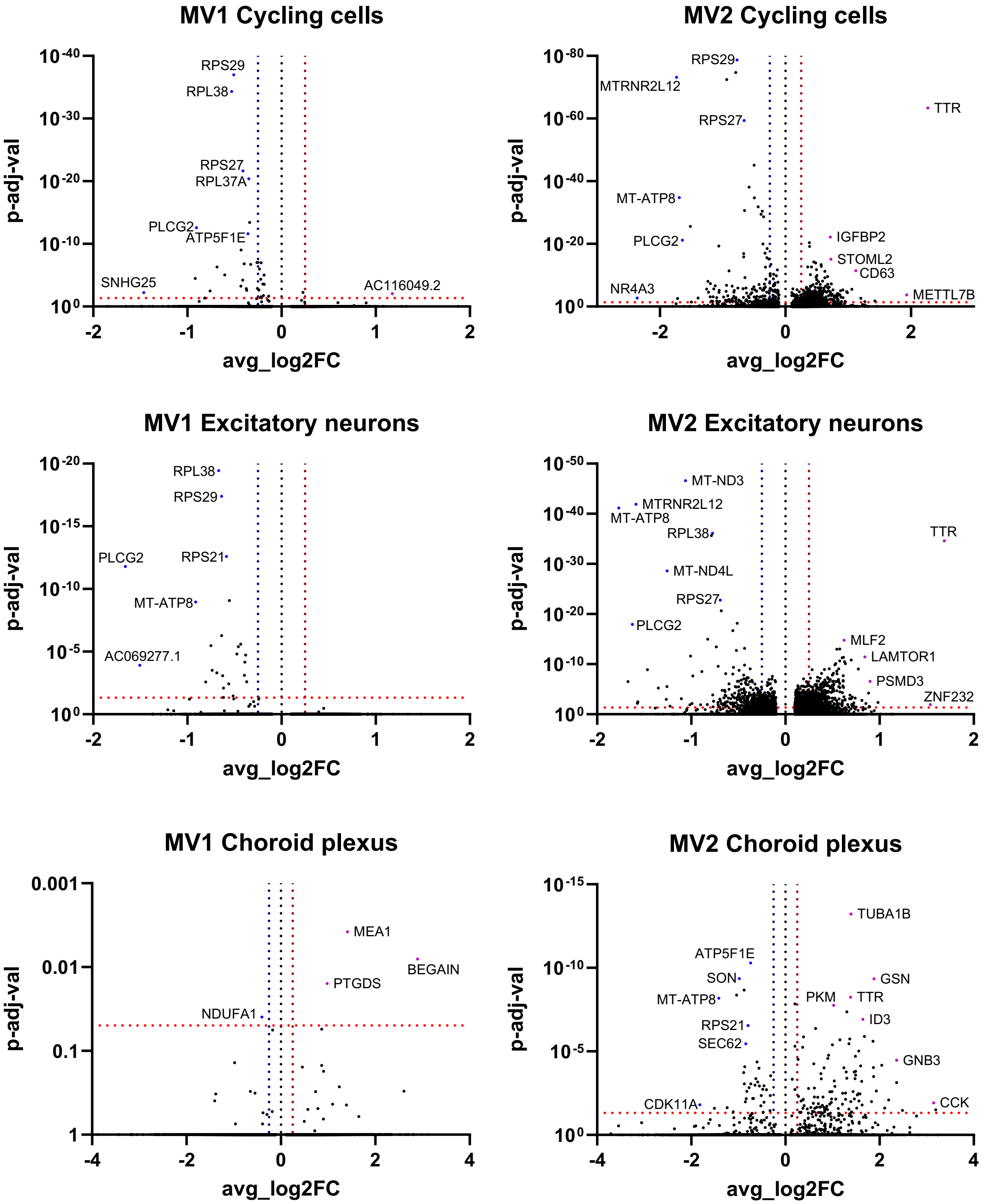
MV1 Cycling cells
RPS29
RPL38
RPS27
RPL37A
PLCG2
ATP5F1E
SNHG25
AC116049.2
MV2 Cycling cells
RPS29
MTRNR2L12
RPS27
TTR
MT-ATP8
PLCG2
IGFBP2
STOML2
CD63
NR4A3
METTL7B
MV1 Excitatory neurons
RPL38
RPS29
PLCG2
RPS21
MT-ATP8
AC069277.1
MV2 Excitatory neurons
MT-ND3
MTRNR2L12
MT-ATP8
RPL38
TTR
MT-ND4L
RPS27
PLCG2
MLF2
LAMTOR1
PSMD3
ZNF232
MV1 Choroid plexus
MEA1
BEGAIN
PTGDS
NDUFA1
MV2 Choroid plexus
TUBA1B
ATP5F1E
SON
GSN
MT-ATP8
PKM
TTR
ID3
RPS21
SEC62
GNB3
CDK11A
CCK
p-adj-val
avg_log2FC

Katie Williams, Bradley R. Groveman, Simote T. Foliaki, Brent Race, Arielle Hay, Ryan O. Walters, Tina Thomas, Gianluigi Zanusso, James A. Carroll, Cathryn L. Haigh. **Two sporadic Creutzfeldt-Jakob Disease subtypes demonstrate common dysfunctional pathways with differing characteristics in vitro.**

Supplemental Figure 9*. Further IHC for neuronal and oligodendrocyte representation in NBH and infected organoids.* IHC and pixel density quantification at 180 dpi of Map2, NF-L and Olig2. Scale bar = 200 µm and all images are shown at the same magnification. The Map2 image for MV1c is a lower magnification of the same region shown in figure 5B.

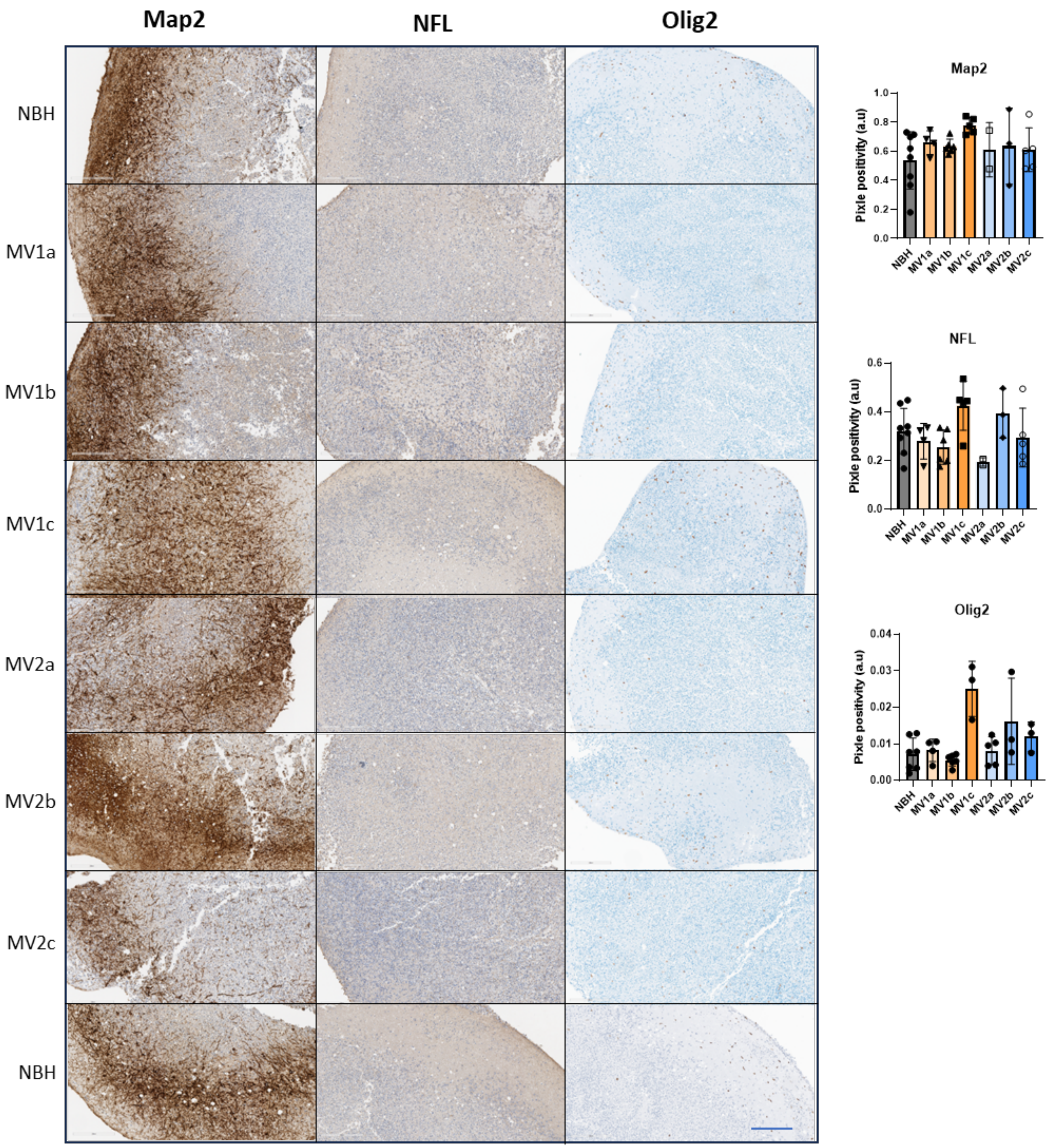

Katie Williams, Bradley R. Groveman, Simote T. Foliaki, Brent Race, Arielle Hay, Ryan O. Walters, Tina Thomas, Gianluigi Zanusso, James A. Carroll, Cathryn L. Haigh. **Two sporadic Creutzfeldt-Jakob Disease subtypes demonstrate common dysfunctional pathways with differing characteristics in vitro.**

Supplemental Figure 10. *PRNP expression across infections and groups.* **A.** UMAP showing log2 detection of *PRNP* transcripts within the NBH and sCJD infected organoids and **B.** Violin plots showing *PRNP* transcript detection across groups. **C.** Violin plots showing *PRNP* transcript detection across the different cell clusters.

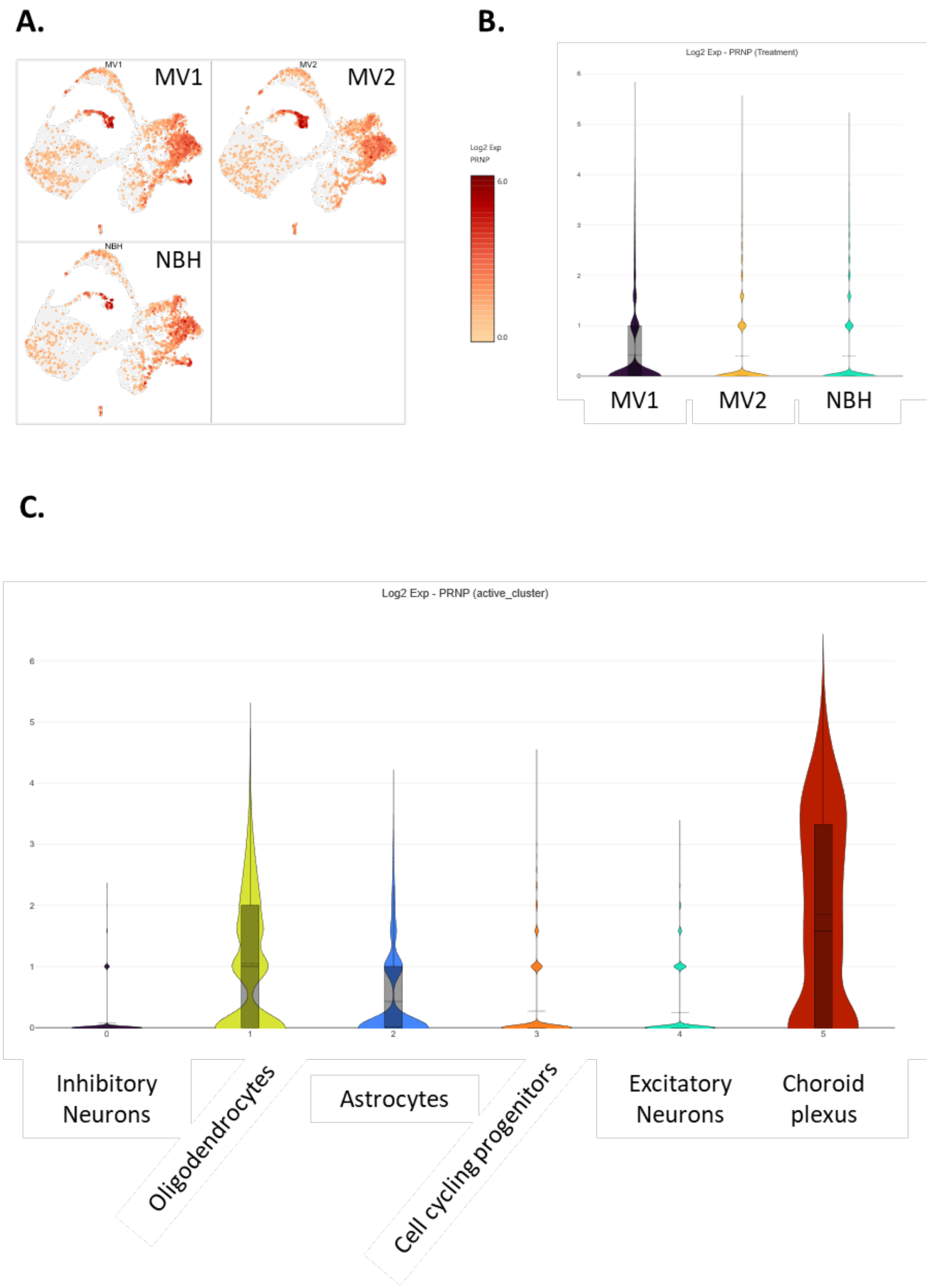

Katie Williams, Bradley R. Groveman, Simote T. Foliaki, Brent Race, Arielle Hay, Ryan O. Walters, Tina Thomas, Gianluigi Zanusso, James A. Carroll, Cathryn L. Haigh. **Two sporadic Creutzfeldt-Jakob Disease subtypes demonstrate common dysfunctional pathways with differing characteristics in vitro.**

Supplemental Figure 11. *Summary of IPA Disease and Biofunctions analysis.* Ingenuity pathway analysis (IPA) comparing the disease and biofunction categories that are changed in the MV1 and MV2 infections from NBH controls across each cell type. The size of the node denotes to -log(B-H p-value) and the colour indicates the IPA prediction of pathway activation (orange), deactivation (blue) or neutral/no calculated activity pattern (grey).

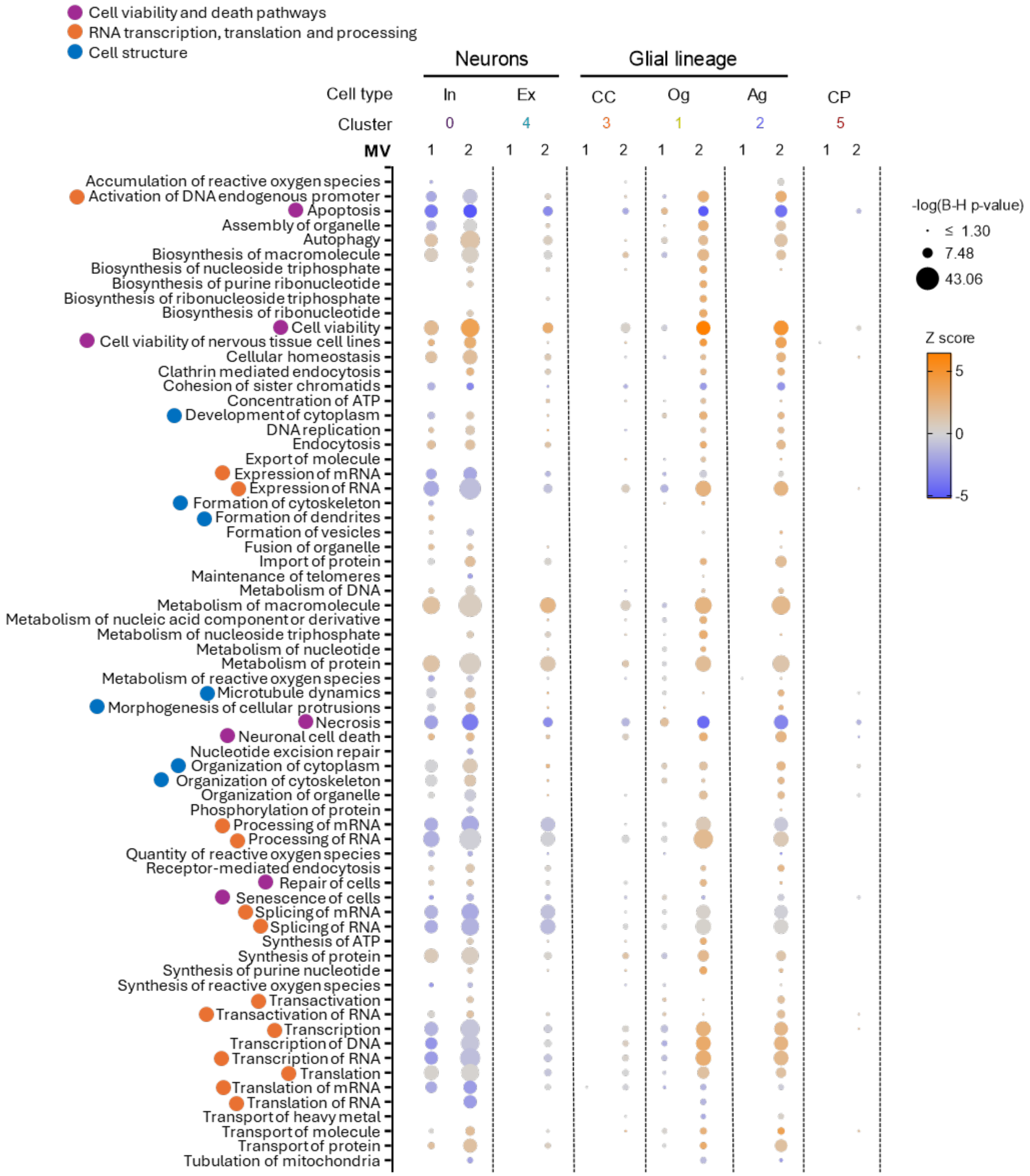

Katie Williams, Bradley R. Groveman, Simote T. Foliaki, Brent Race, Arielle Hay, Ryan O. Walters, Tina Thomas, Gianluigi Zanusso, James A. Carroll, Cathryn L. Haigh. **Two sporadic Creutzfeldt-Jakob Disease subtypes demonstrate common dysfunctional pathways with differing characteristics in vitro.**

Supplemental Figure 12. *Human brain inocula.* Western blotting of the inoculums used for infecting the organoids following proteinase K digest to reveal the banding pattern of the PrP. PrP was detected using the 3F4 antibody.

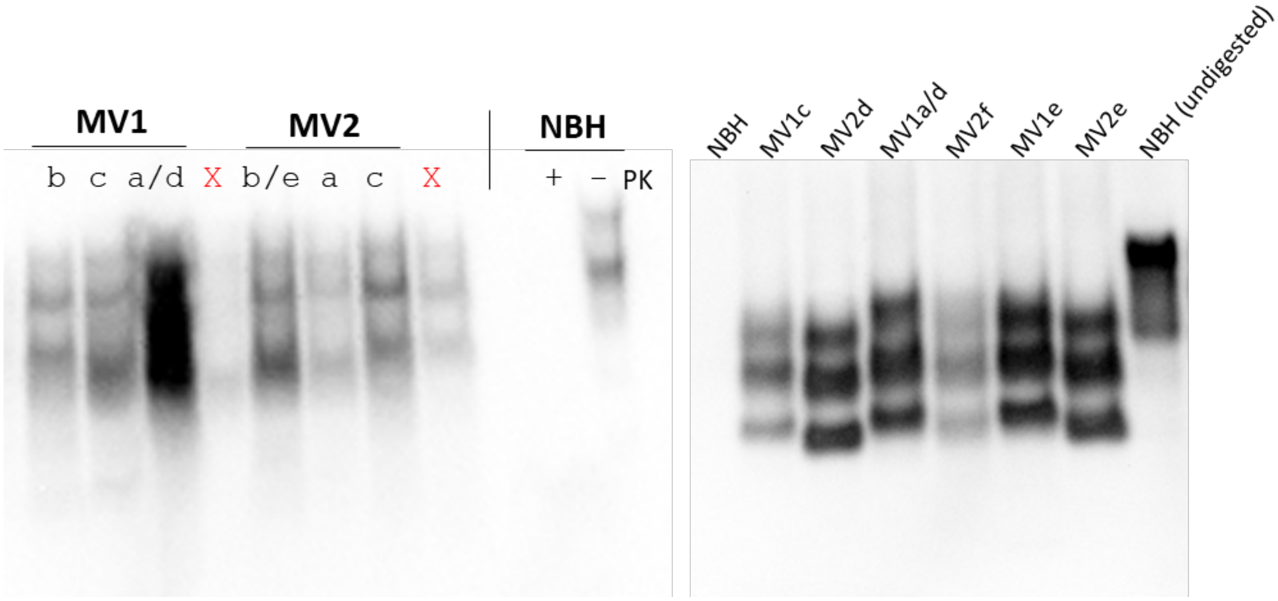

Supplement: Supplemental data [file jci-136-194721-s316.pdf]
