# Supplementary material for: Distinct neuronal alterations distinguish two subtypes of sporadic Creutzfeldt-Jakob disease with shared dysfunctional pathways
Source: J Clin Invest. 2026 Feb 12;136(8):e194721. doi: 10.1172/JCI194721 (PMC13078861; doi:10.1172/JCI194721)

Full unedited blot images for Figure 1 – PrP. All lanes were used unless otherwise indicated.

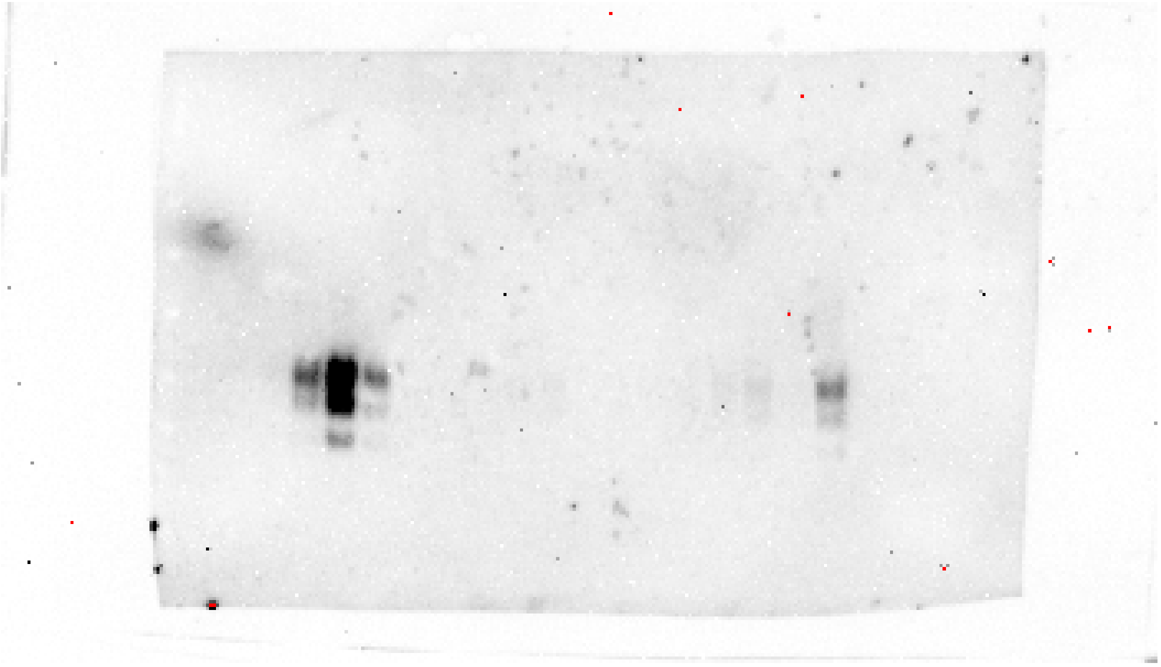

Not used in this study

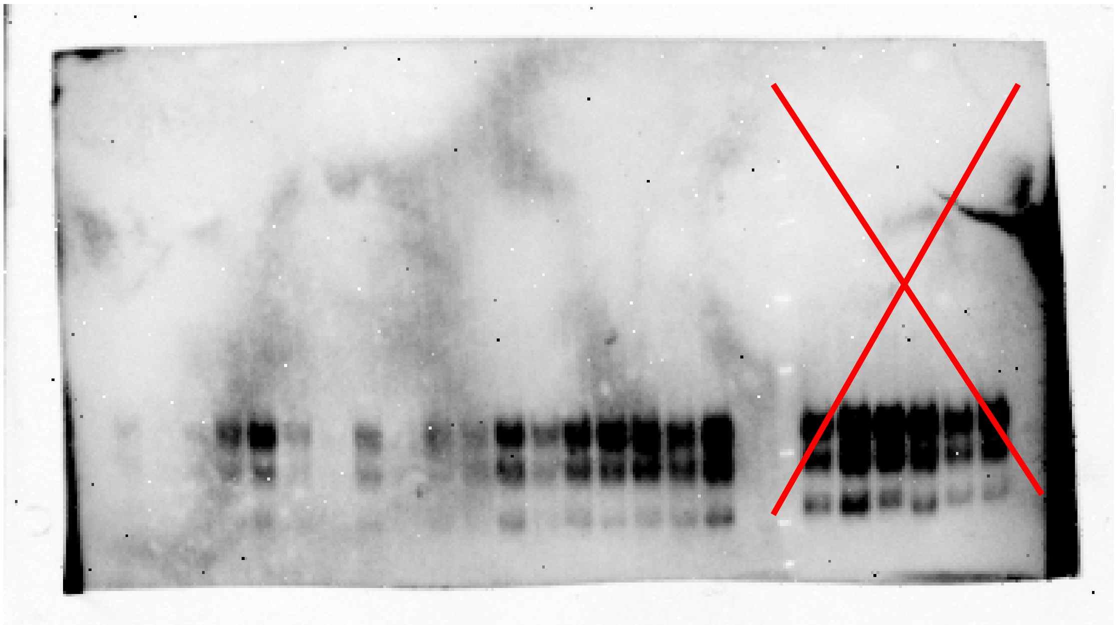

Full unedited blot images for Figure 2 – PSD95. All lanes were used.

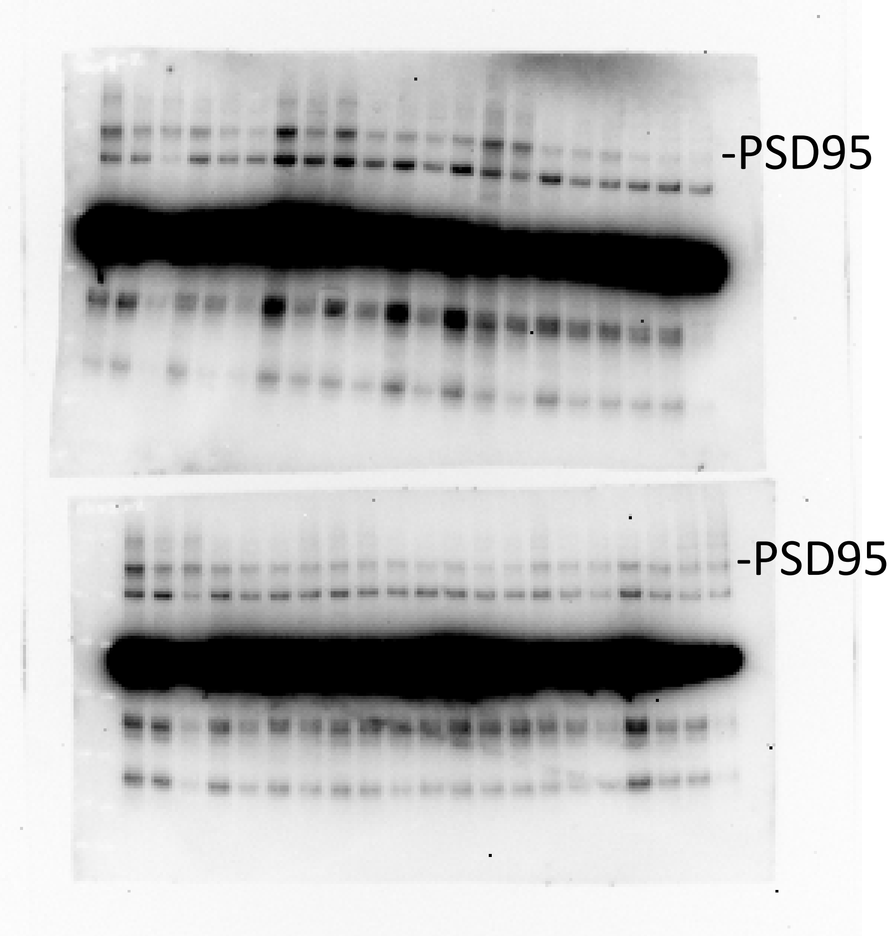

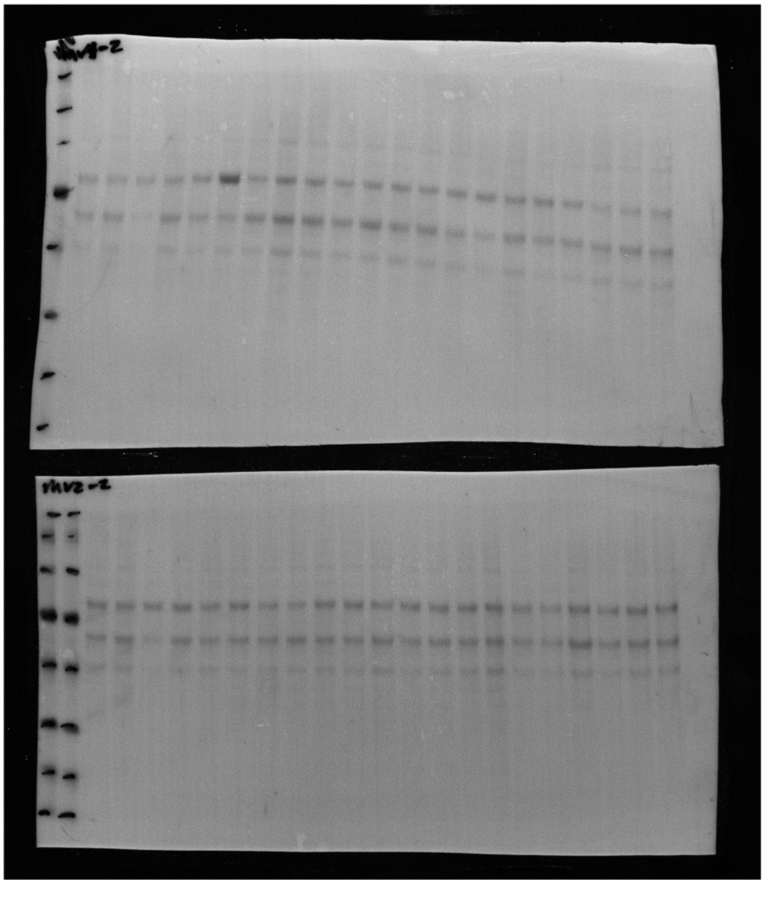

Full unedited blot images for Figure 3 – TOMM20. All lanes were used.

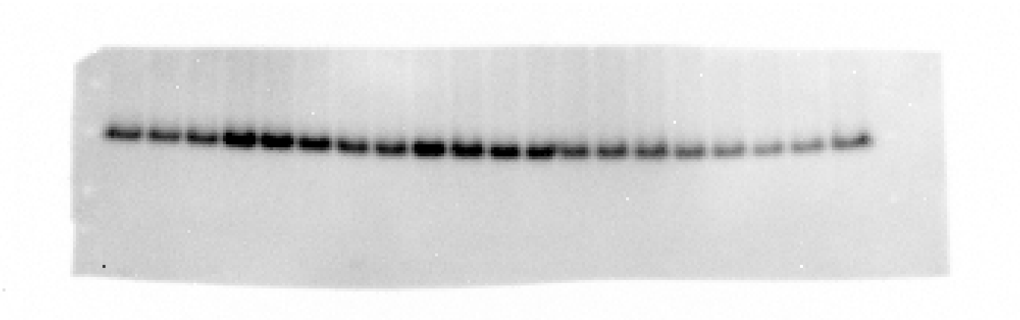

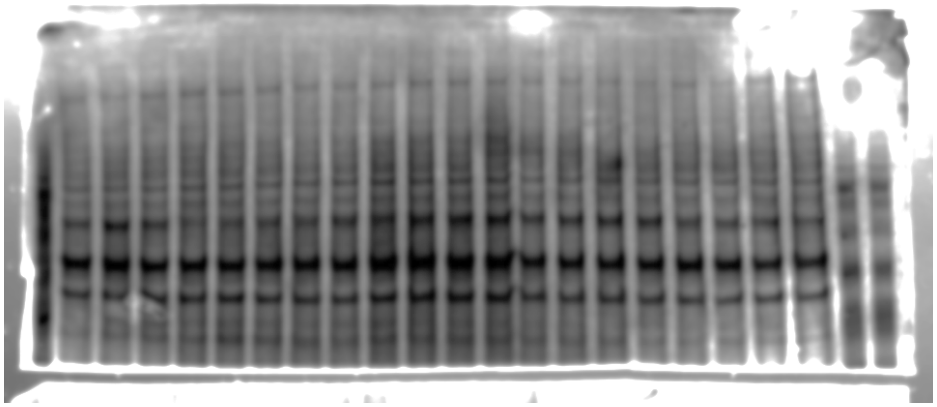

These blots were not shown in the figure but were included in the quantification analysis

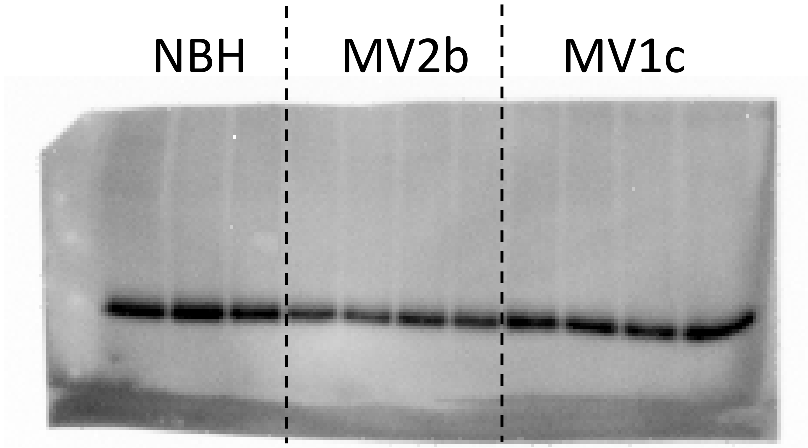

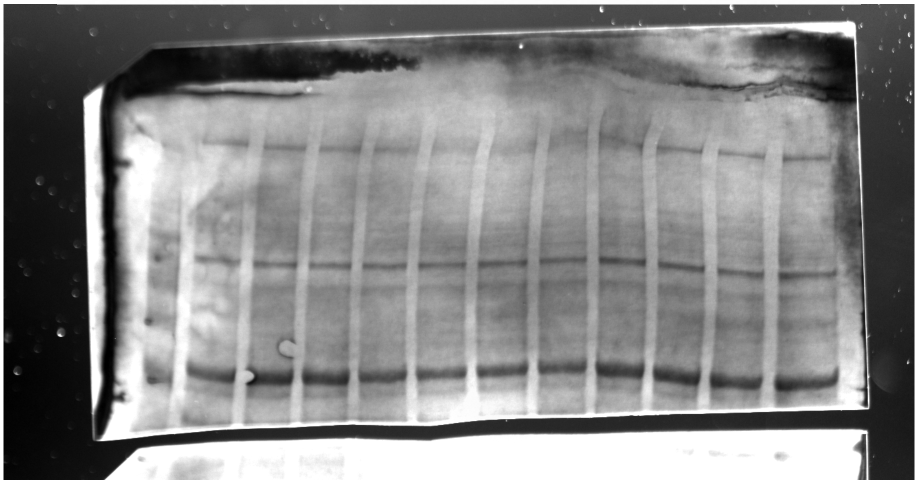

Full unedited blot images for Figure 3 – p62 & LC3. All lanes were used.

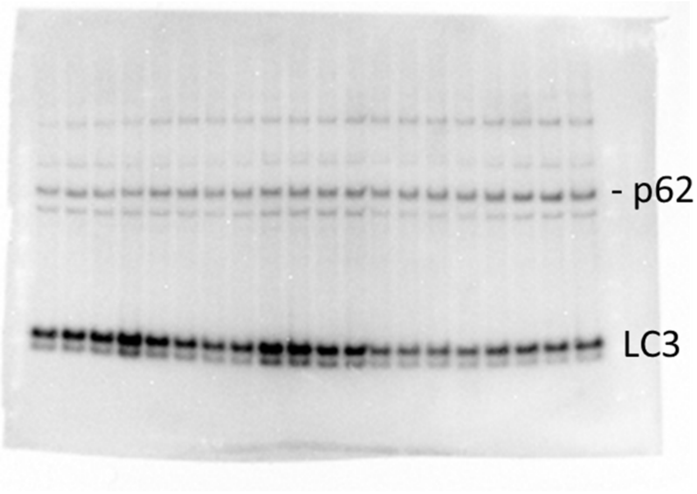

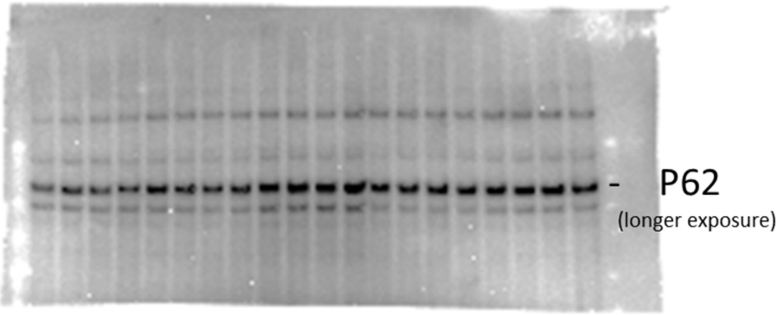

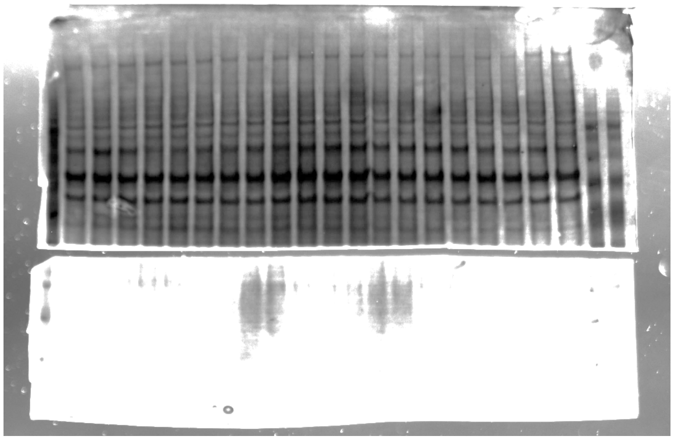

These blots were not shown in the figure but were included in the quantification analysis

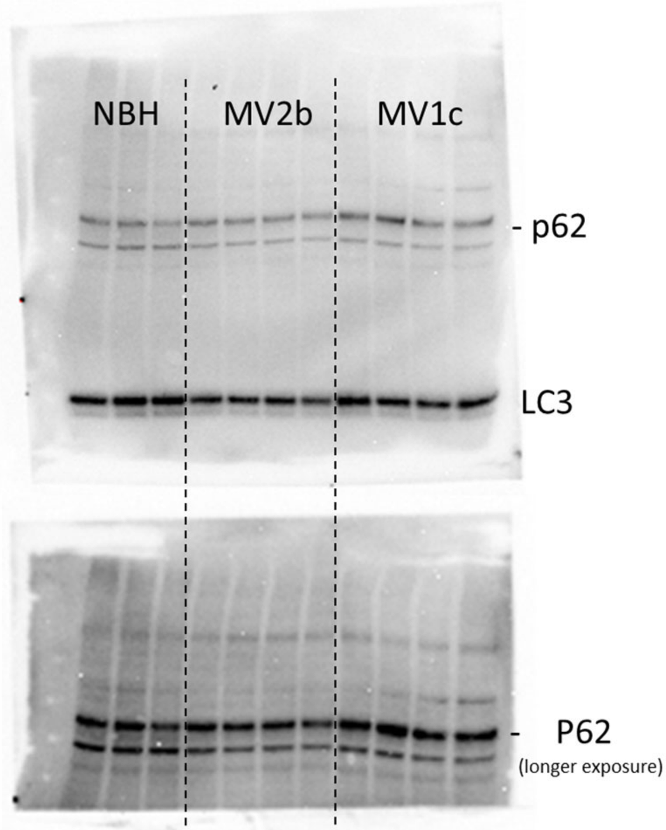

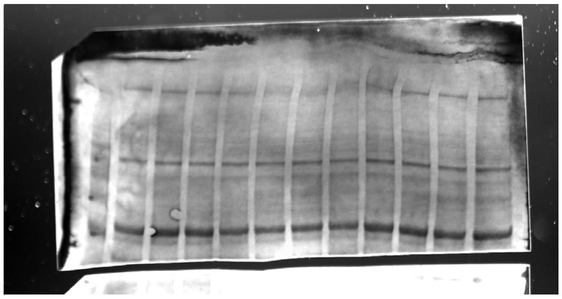

Full unedited blot images for Figure 5 – NF-L. All lanes were used.

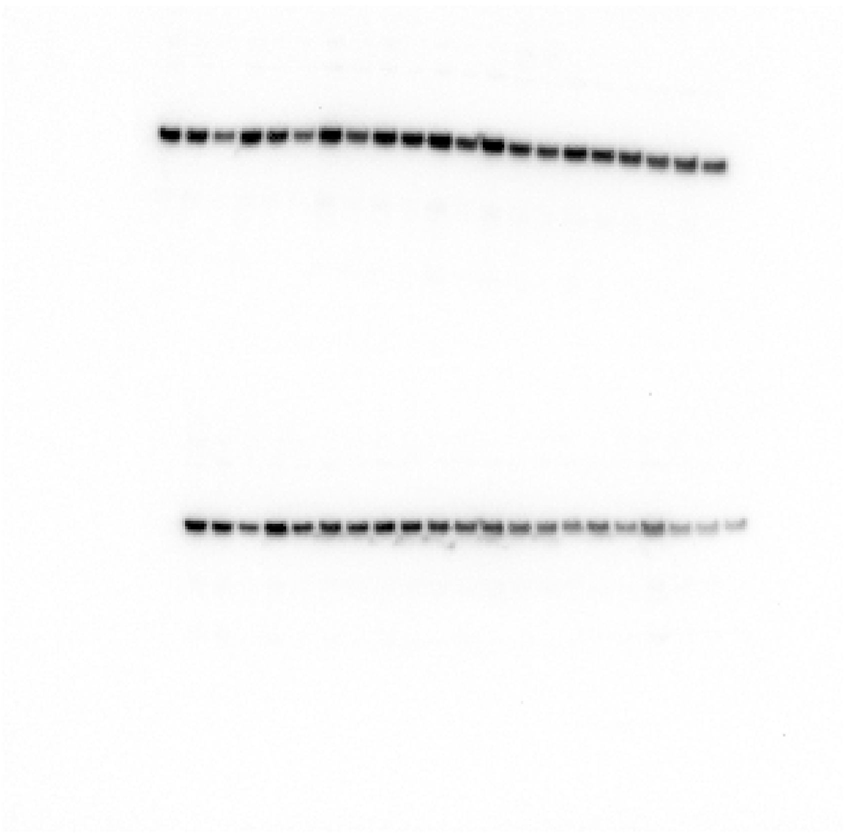

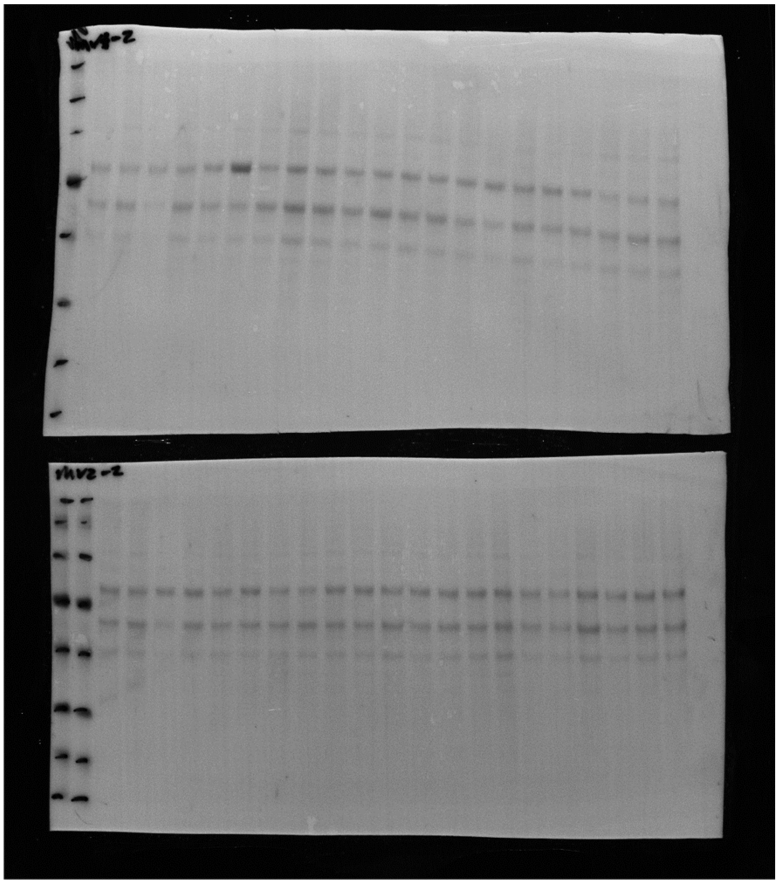

Full unedited blot images for Supplementary File 1 – PrP. All lanes were used.

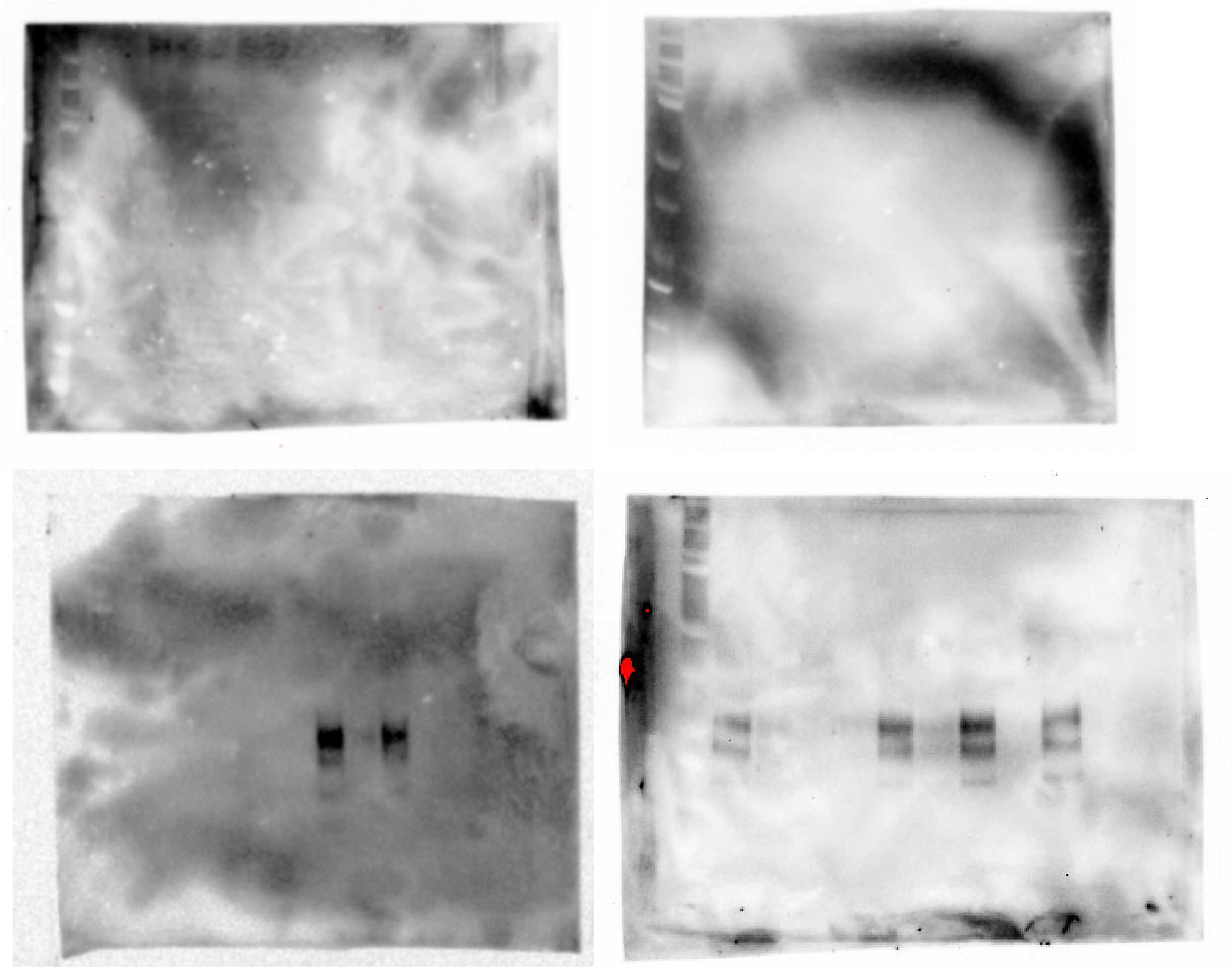

Full unedited blot images for Supplementary File 2 – GFAP. All lanes were used.

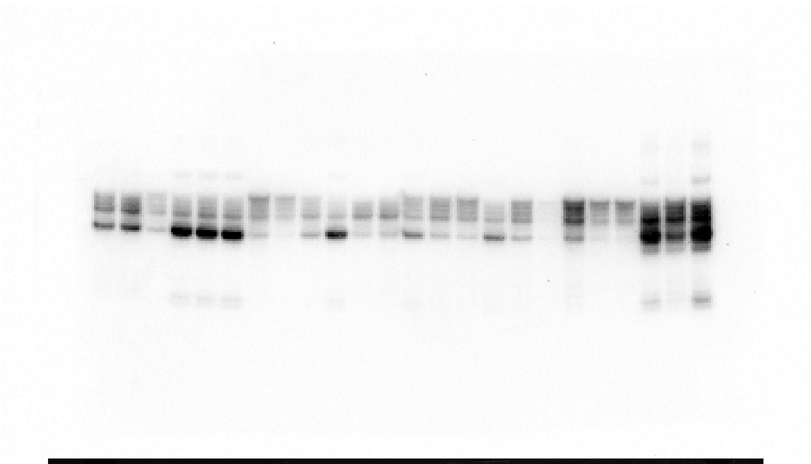

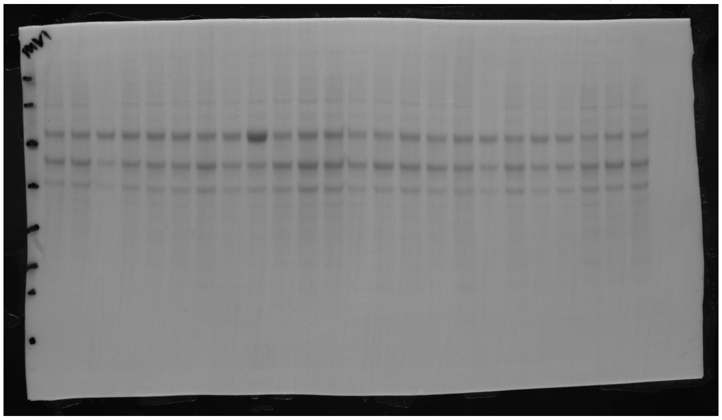

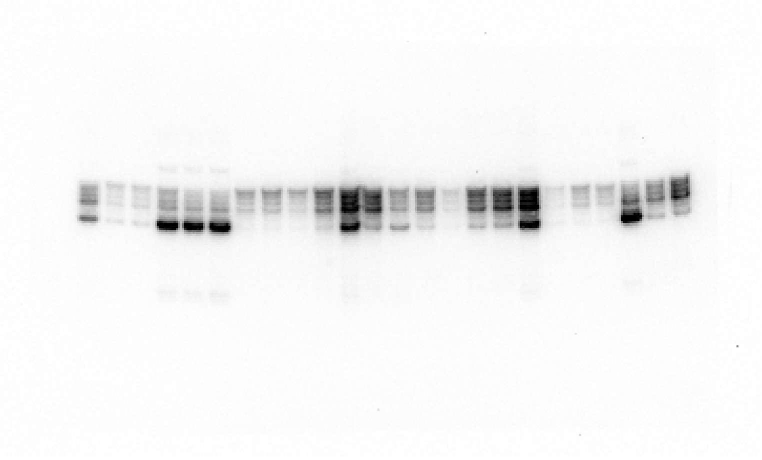

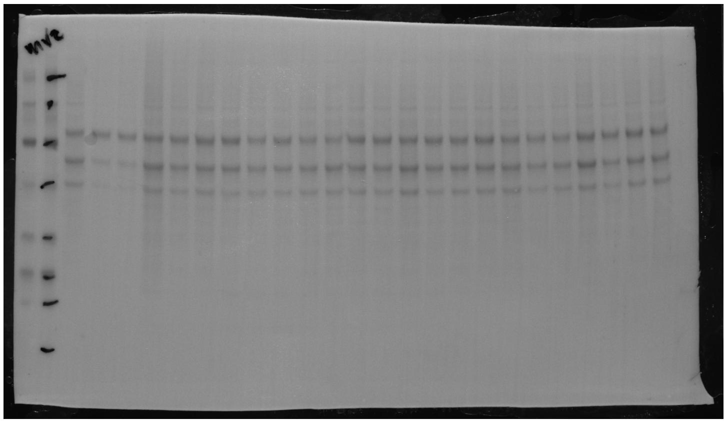

Full unedited blot images for Supplementary File 8 – pEGFR/EGFR. All lanes were used.

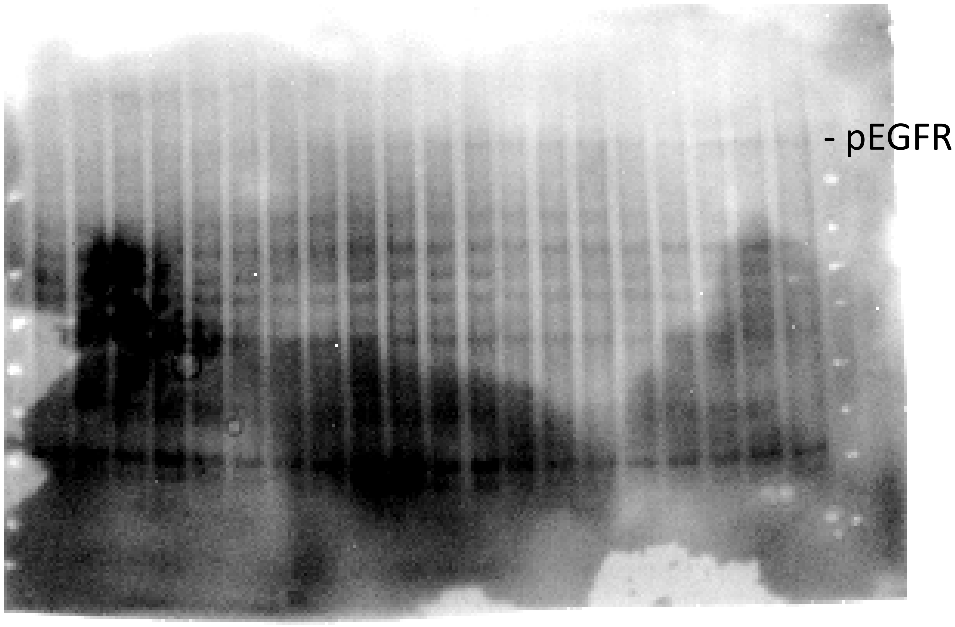

- pEGFR

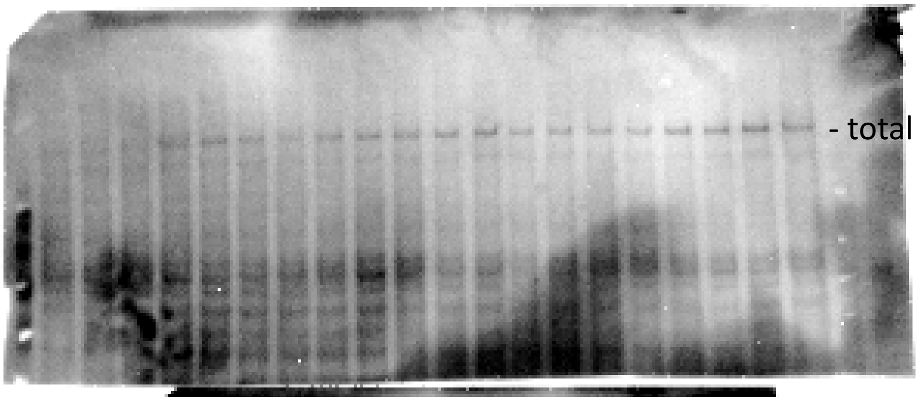

- total EGFR

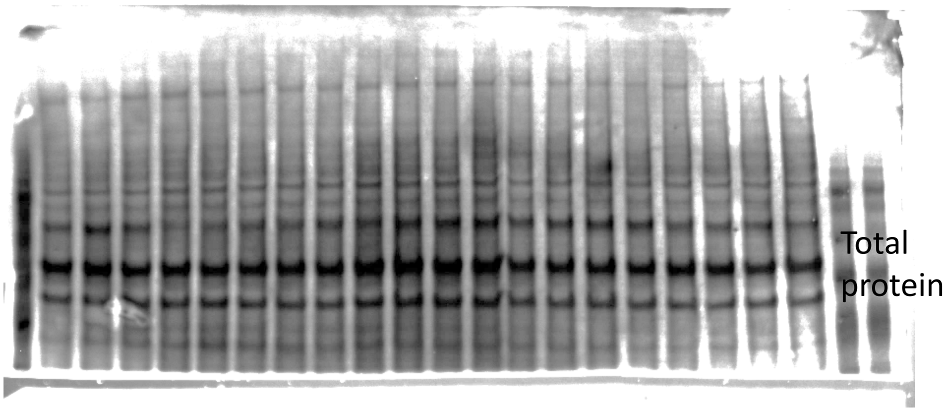

Total protein

Full unedited blot images for Supplementary File 8 – pEGFR/EGFR.
All lanes were used.

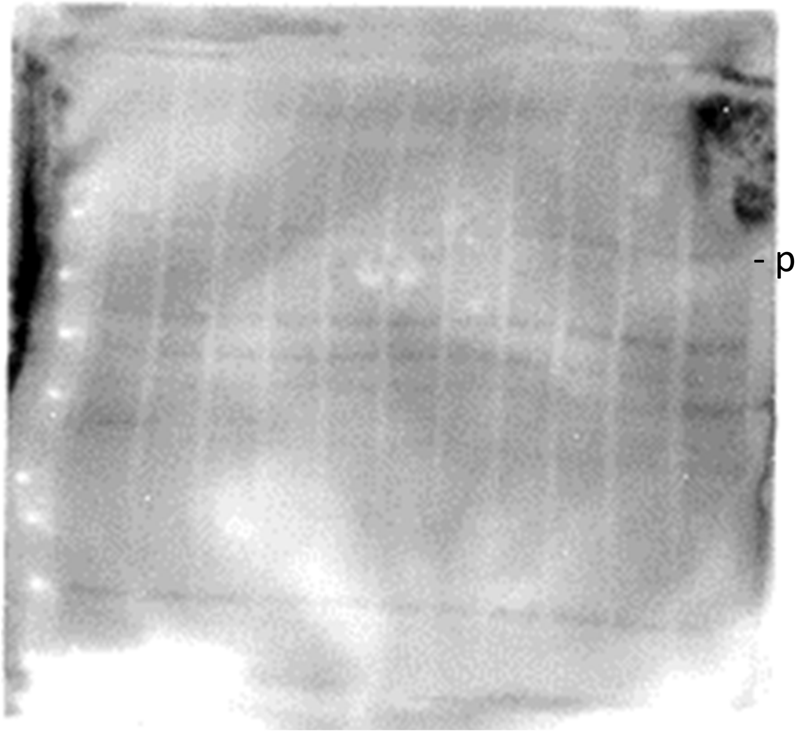

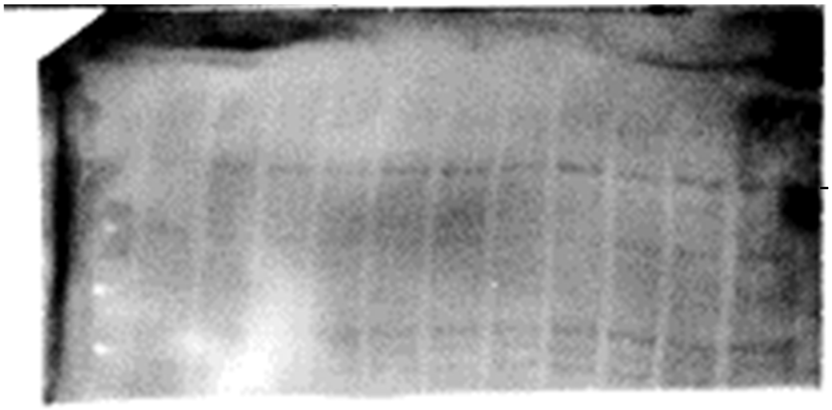

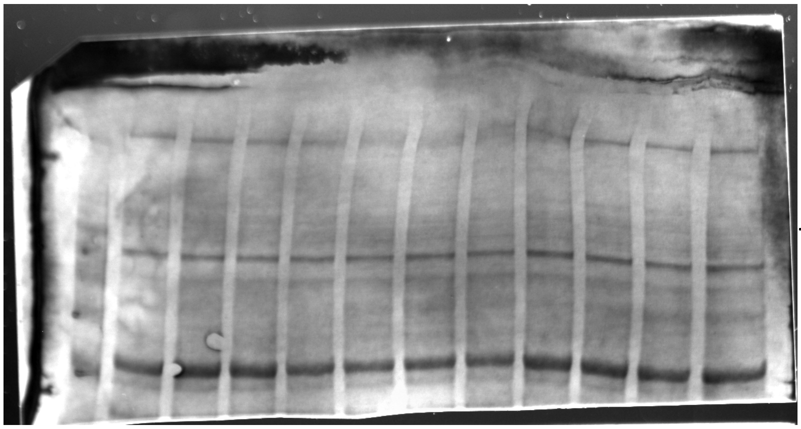

Full unedited blot images for Supplementary File 15 – PrP. Lanes not used are indicated.

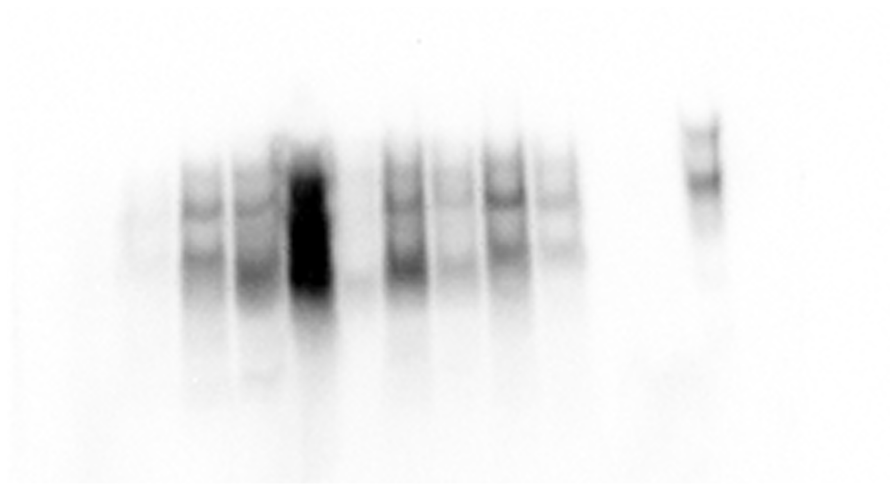

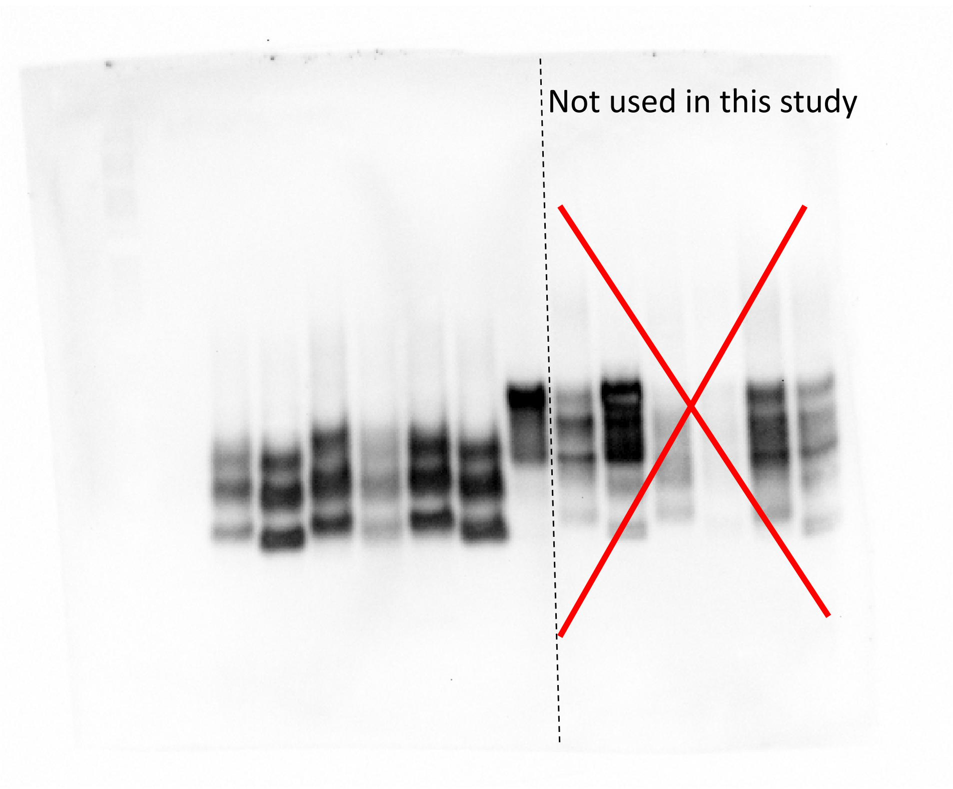

Supplement: Unedited blot and gel images [file jci-136-194721-s317.pdf]
